# Comparison of Five Modeling Approaches to Quantify and Estimate the Effect of Clouds on the Radiation Amplification Factor (RAF) for Solar Ultraviolet Radiation

**DOI:** 10.3390/atmos8080153

**Published:** 2017

**Authors:** Eric S. Hall

**Affiliations:** National Exposure Research Laboratory, Office of Research and Development, US Environmental Protection Agency, Mail Drop E205-03, 109 T. W. Alexander Drive, Research Triangle Park, NC 27711, USA

**Keywords:** Radiation Amplification Factor (RAF), solar zenith angle (SZA), Dobson Unit (DU), ultraviolet (UV), ultraviolet radiation (UVR), cloudiness

## Abstract

A generally accepted value for the Radiation Amplification Factor (RAF), with respect to the erythemal action spectrum for sunburn of human skin, is −1.1, indicating that a 1.0% increase in stratospheric ozone leads to a 1.1% decrease in the biologically damaging UV radiation in the erythemal action spectrum reaching the Earth. The RAF is used to quantify the non-linear change in the biologically damaging UV radiation in the erythemal action spectrum as a function of total column ozone (O_3_). Spectrophotometer measurements recorded at ten US monitoring sites were used in this analysis, and over 71,000 total UVR measurement scans of the sky were collected at those 10 sites between 1998 and 2000 to assess the RAF value. This UVR dataset was examined to determine the specific impact of clouds on the RAF. Five de novo modeling approaches were used on the dataset, and the calculated RAF values ranged from a low of −0.80 to a high of −1.38.

## 1. Introduction

The Radiation Amplification Factor (RAF) is defined as the measured percentage change in ultraviolet (UV) irradiance for each one-percent change in total column ozone [1]. Understanding variations in measured ultraviolet radiation (UVR) over time, along with associated RAF values, assists health scientists in determining risks associated with UVR exposure through the RAF for individuals and the environment [2]. Exposure to ultraviolet radiation (UVR) poses risks to both humans and the environment, therefore a number of national and international government, industrial, and university research organizations initiated programs to measure the amount of UVR reaching the Earth’s surface, since stratospheric ozone affects the amount of UVR reaching the Earth. The United States Environmental Protection Agency (US EPA) conducted a research program from 1996 to 2004 to measure ultraviolet radiation (UVR) at 21 network sites throughout the continental US, Alaska, Hawaii and the US Virgin Islands (St. John) under all weather conditions. The US EPA National Exposure Research Laboratory (NERL) developed the UV Radiation Research Program to measure intensity of UVR at distinct locations throughout the US that differed in spatial location, geography, climate, altitude, and ecology. The three major categories of research-grade instruments that have been used to detect UVR are broadband, narrowband, and spectral. NERL chose spectral UVR monitors called Brewer Spectrophotometers. When operated within a disciplined calibration and maintenance program, the instruments precisely measure UVR levels through a well-defined portion of electromagnetic spectrum wavelengths (286.5 nm to 363 nm). Maintaining long-term calibration of spectral instruments requires great effort. They are more expensive to operate in comparison to broadband or narrowband instruments, because they require highly skilled operators.

The Brewer instruments were calibrated for biologically damaging UV radiation in the erythemal action spectrum at each of the 21 sites using a secondary (travelling) standard lamp traceable to a primary (stationary) National Institute of Standards and Technology (NIST) 1000-W lamp. The calibrations were performed by scientists at the University of Georgia at Athens (UGA) National UV Monitoring Center (NUVMC) on an annual basis. The calibrated Brewer data used a daily temporal response (estimated) based on the annual calibration. In addition, independent quality assurance audits of the Brewers were performed by scientists from the National Oceanic and Atmospheric Administration (NOAA) Central UV Calibration Facility (CUCF). Quarterly checks on the transfer of the calibration standard from the NIST 1000-W lamp to the traveling secondary standard were performed at the NUVMC. The response function of each instrument was calculated daily from a linear interpolation between the two (temporally) closest response functions. Brewer data were corrected for dark count, dead time, and stray light.

Clouds affect the relative absorption/reflectance of UVR. Transmittance of UVR through clouds is shown to be wavelength dependent, ranging from 45% in the UV-A region to 60% in the UV-B region as stated in Chapter 4, page 105 of [3]. Cloud features known to affect the transmission of UVR include cloud amount and coverage (e.g., percentage of sky covered), particle size distribution, cloud spatial and temporal variability, season, location, etc., [4]. Realistically, clouds can either increase or decrease the amount of UVR at the surface [5]. Due to the unpredictable nature of clouds, the extent of cloudiness (percent clearness) of the sky had to be determined for this analysis in a consistent, logically defensible manner, to determine the effect of clouds on the Radiation Amplification Factor (RAF). Using a consistent definition of cloudiness, the results seem to indicate that average RAF values generally approximate −1.1 at these 10 sites (two urban sites and eight US National Parks sites), and that total column ozone (O_3_) and solar zenith angle (SZA) are the most important parameters in determining the biologically damaging UV radiation in the erythemal action spectrum reaching the Earth’s surface. Five modeling approaches, labelled A, B, C, D, and E, were used to estimate the impact of clouds on the biologically damaging UV radiation in the erythemal action spectrum.

### 1.1. Development of the EPA’s UVR Monitoring Network

**Figure 1:**
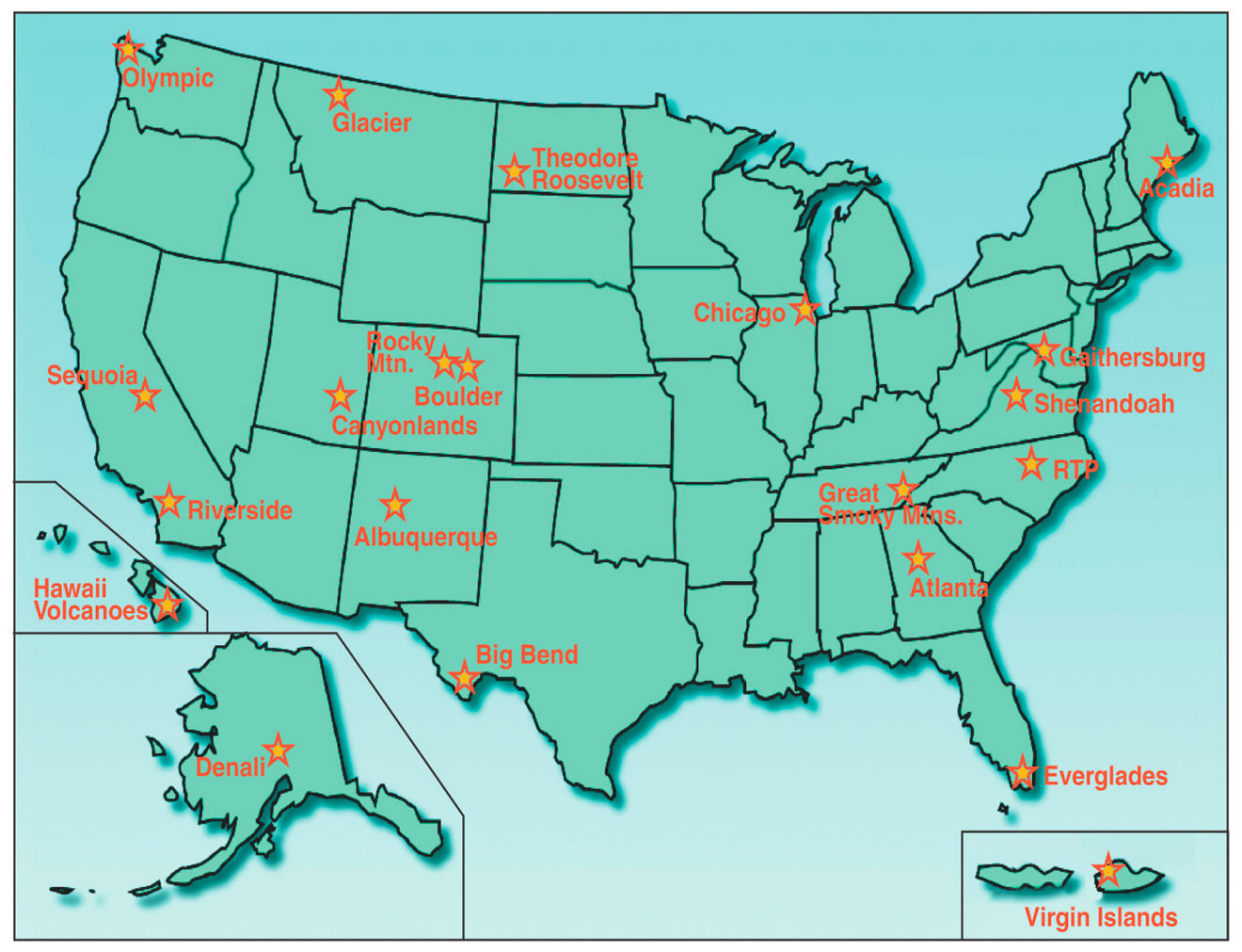
Location of Brewer Spectrophotometers in the Environmental Protection Agency (EPA) ultraviolet radiation (UVR) Monitoring Network.

In the early 1990s, the EPA’s Office of Research and Development (ORD) originally designed a UVR monitoring network of five to seven sites principally in urban areas, with a few “pristine” rural sites for monitoring background UVR levels. The EPA’s role in UVR research was to monitor in urban areas. The United States Department of Agriculture (USDA) was responsible for monitoring UVR in rural areas. The focus of the EPA’s original UVR network design was shifted in 1996. In September 1996, the US EPA and US National Parks Service (NPS), of the US Department of the Interior (DOI), signed an Interagency Agreement (IAG) to cooperate on a program of long-term monitoring of environmental stressors, including UVR, at 21 separate locations throughout the United States. When the NPS sites were added to the network, it greatly expanded the EPA’s UVR monitoring program. The EPA’s UVR Monitoring Network facilitated research on the observed effects that environmental stressors, including UVR, pose on various ecosystems and on human health. The 21 EPA UVR monitoring sites (displayed in Figure 1) were located throughout the continental United States, Alaska (Denali National Park), Hawaii (Hawaii Volcanoes National Park) and the US Virgin Islands (Virgin Islands National Park, St. Johns, VI). Fourteen of the sites were located in US National Parks, and seven sites were located in urban settings. Data collected from the 21 sites was processed through a quality assurance protocol to ensure proper characterization of UVR intensities measured at each site.

The 21 deployed UVR monitoring instruments measured full sky UV radiation. These instruments tracked the sun and monitored the variation in solar energy throughout the day. The instruments measured UVR at wavelengths between 286.5 nm and 363 nm in 0.5 nm wavelength increments [6]. Therefore, each scan produced 154 discrete wavelength values for which UVR would be measured. The data collected at each network site could then be used to calculate both the dose and dose rate of UVR received at the Earth’s surface at various times throughout the day. The instruments were mounted on an azimuth tracker and tripod unit connected to a desktop computer running the Disk Operating System (DOS) and GW^™^ Basic Software for instrument control.

The major objectives of the EPA’s UVR Monitoring Network were to:

1. Improve understanding of the nature and intensity of UVR reaching the Earth’s surface.
2. Characterize the physical and chemical parameters that modify UVR flux.
3. Obtain better estimates of UVR exposures at multiple times, locations, meteorological conditions, altitudes, terrain characteristics, topologies, and air pollution conditions.
4. Evaluate human and ecosystem/environmental exposure to surface UVR across the US.
5. Assess the impact of changes in stratospheric ozone and tropospheric pollution on biologically damaging UV radiation in the erythemal action spectrum.
6. Assess the effectiveness of control strategies (e.g., Montreal Protocol) designed to reduce the amount of greenhouse gases in the stratosphere, and increase the amount of stratospheric ozone.
7. Serve as a component of the EPA’s National Environmental Monitoring Strategy.

During the development phase of the EPA’s UVR Monitoring Network, the experimental design and site selection were planned to test specific hypotheses, which fulfilled the stated research objectives listed above. Sites were selected which provided UVR data for a variety of conditions (e.g., high and low elevation, high and low cloud cover, high and low air pollution levels, latitude and longitude variation, ecosystem variations [fresh and marine water], etc.). The EPA decided that the network would consist of spectral UVR monitors so that researchers could determine what key factors caused changes in UVR levels, e.g., changes in stratospheric ozone composition, tropospheric changes like cloud cover or pollution (e.g., black carbon, and particulate matter), etc. A secondary benefit of spectral UVR data is that the data on the UV spectrum can be compared with data on human and ecological disease incidence and geographic distribution.

The first (experimental) site in the EPA’s UVR Monitoring Network at Research Triangle Park, North Carolina, began initial operation in 1992. The UVR data collected from the EPA’s UVR Monitoring Network was processed through a Level-1 data quality assurance algorithm to ensure proper characterization of biologically damaging UV radiation intensities at each measured wavelength (for each instrument), and the data in this study is designated as Level-1 corrected data [7]. The Level 1 algorithm included corrections for calibration drift of the instruments, cosine response, and temperature effects. Once the raw UVR monitoring data is run through the L1 algorithm, the acceptance or rejection of a scan measurement value is based on the number of successful instrument scans during a given day and the range of the L1-corrected biologically damaging UV radiation values. During the term of the monitoring program, a total of 40,474 site-days’ worth of UVR data was collected through the EPA’s UVR Monitoring Network, with 35,811 site-days being usable for analysis (88.5%). The network collected over 500,000 individual measurements (scans) of UVR data during the program [8].

### 1.2. Importance of UVR Monitoring

The sun is a near-ideal blackbody radiator with a temperature of 6000 degrees (6000°) Kelvin, emitting electromagnetic radiation in a wide and continuous spectral distribution; however, spectral instruments with photo diode arrays or charge-coupled devices (CCDs) can record the entire spectrum simultaneously [9]. The wavelength peak in its emission curve resides in the visible wavelength region. Although the sun emits a tremendous amount of solar energy on a daily basis, the Earth captures only a small portion of the sun’s radiant energy, approximate 1370 Watts per meter squared (W/m^2^) per day [10]. The energy in the UV-A portion of the electromagnetic spectrum is approximately 6.3% of the total solar energy received at the edge of the Earth’s atmosphere on a daily basis (wavelengths: 315–400 nm), while the UV-B (wavelengths: 280–315 nm), and UV-C (wavelengths: 200–280 nm) portions represent 1.5% and 0.5% respectively [11]. Approximately 9% of the sun’s total solar energy output is in the UV spectrum. A high percentage of UV-A reaches the Earth’s surface (because it is weakly absorbed by atmospheric ozone), but has a low impact on biological systems. UV-B has a greater impact on biological systems than UV-A, but is efficiently screened by ozone. The EPA’s UVR monitoring program was conducted in an attempt to detect trends in UVR measurements over time across a wide geographic area.

### 1.3. Stratospheric Ozone

The UVR flux is affected by changes in the amount of stratospheric ozone (O_3_) [12]. The amount of UVR reaching the surface of the Earth is increased when there is a decrease in the amount of stratospheric ozone. One of the major factors contributing to a decrease in stratospheric ozone is the use of chlorofluorocarbons (CFCs) [13]. CFCs in the stratosphere react with ozone through photochemical reactions, which decompose ozone into both monomolecular oxygen and diatomic oxygen species, and although the amounts of CFCs used have been significantly reduced, these compounds remain in the atmosphere for durations of up to 40–60 years, contributing to stratospheric ozone reduction [14]. The atmospheric lifetimes of CFCs can be extremely long. CFC-12 (dichlorodifluoromethane), CCl_2_F_2_, has an atmospheric lifetime of 100 years, while HCFC-22 (chlorodifluoromethane), CHClF_2_, has an atmospheric lifetime of approximately 14.6 years [15]. As stratospheric ozone is reduced, there is less ozone available to absorb UVR, resulting in increased amounts of UVR reaching the Earth’s surface (holding all other factors constant). Measurement of UVR intensities across the United States assisted US policymakers in assessing the effectiveness of the Montreal Protocol [16], in reducing the important ozone-depleting substances such as CFCs. Title VI of the Clean Air Act [17] also governs the protection of stratospheric ozone.

## 2. Materials and Methods

### 2.1. Measurement of the Ultraviolet (UV) Spectrum

Spectral instruments measuring UVR are called scanning spectroradiometers, and these instruments make continuous, spectrally resolved measurements either across the entire electromagnetic spectrum or specific portions of it. Most spectral instruments contain photomultiplier detectors with either single or double monochromators, where light passes through an initial diffraction grating (usually 1200 or 2400 lines per nm) and through a middle slit which directs the light onto a second diffraction grating. The multiple grating configuration minimizes stray light from adjacent wavelengths caused by the rapid change of UVR intensity at wavelengths below 320 nm. Multiple diffraction gratings also improve the wavelength resolution of scanning spectroradiometers, which can be as low as 0.5 nm [18]. It usually takes several minutes for scanning spectroradiometers to make a single complete scan. The Brewers used in the EPA’s network were spectral instruments requiring approximately six min to complete a single scan, which introduced temporal variability when clouds pass overhead during a scan period.

### 2.2. UVR Monitoring Instruments

**Figure 2:**
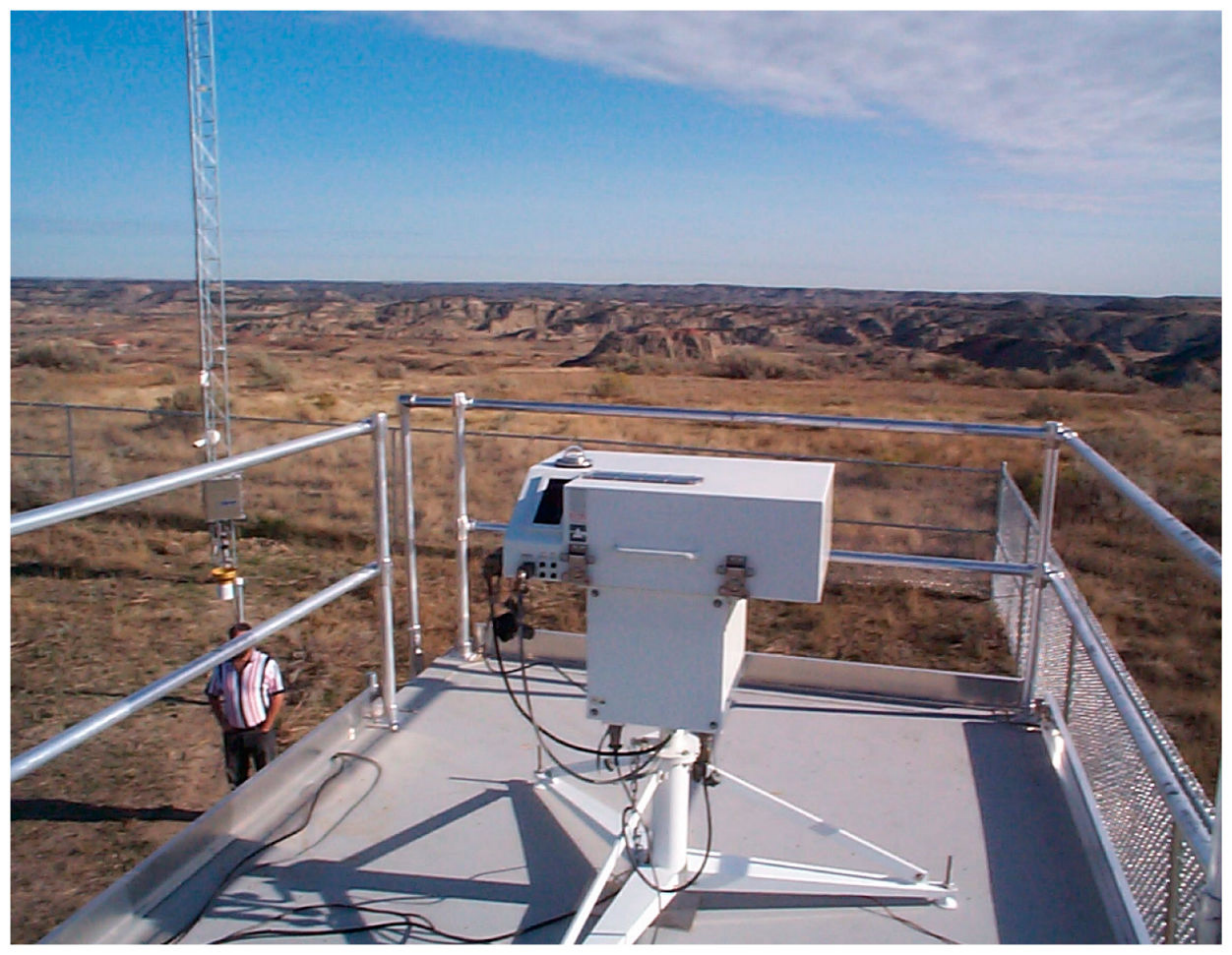
Brewer Spectrophotometer located in Theodore Roosevelt National Park, North Dakota.

The Brewer is manufactured by Kipp and Zonen (formerly SCI-TEC, Saskatoon, SK, Canada). Figure 2 displays a Mark III Brewer Spectrophotometer that was located in Theodore Roosevelt National Park in North Dakota as part of the EPA’s UVR Monitoring Network. Brewers measure UVR in wavelengths ranging from 286.5 nm to 363 nm. The measurement resolution for this device is 0.5 nm. This means that 154 separate UVR values are recorded, one value for each wavelength, during each full scan of the instrument. Brewers complete 22 to 39 measurement scans per day during summer daylight hours depending on the latitude of the instrument, with 32 measurement scans being the minimum number of ideal scans per day for each instrument during that period. Throughout a full year, the expected number of daily scans is approximately 15–20. Normal instrument repair and maintenance caused by components such as its nickel sulfate filter, photomultiplier tube, bearings, and surges due to lightning strikes and power interruptions reduced instrument availability and subsequently the number of scans. The device’s scan rate is controlled by ephemeris scheduling software, taking more UVR measurement scans as solar noon approaches at its particular location. The recorded UVR data is stored in the computer and archived for post-processing through an algorithm that checks for inconsistent data and other anomalies.

The instrument computer controls the azimuth tracker which rotates the entire Brewer, allowing it to track the sun. Brewer software has an ephemeris algorithm, which calculates both the azimuth and zenith angles of the sun as seen from its (current) location. Brewers accomplish this using its latitude and longitude placement along with its GMT time and date. Angles calculated by the ephemeris algorithm along with latitude and longitude are transmitted to both the zenith tracker and azimuth tracker systems to properly position the Brewer towards the sun. Brewers measure biologically damaging UV radiation at each wavelength (spectral irradiance) in the instrument’s measurement range during each scan of the instrument. Spectral irradiance is expressed as power density per unit wavelength (i.e., watts [or milliwatts] per square meter per nanometer [mW/m^2^/nm]). UVR data collected by the instruments is important because it helps us understand the implications of increased UVR associated with decreasing stratospheric ozone concentrations. The instruments provide accurate UVR measurements of known quality in assessing the effectiveness of the EPA’s stratospheric ozone policies. The data allows scientists to evaluate the “climatology” affecting UVR in the environment. The EPA’s quality-assured UVR data is posted to a publicly accessible web site: https://www.epa.gov/hesc/rsig-data-inventory (Last Accessed: 4 August 2017).

Each instrument in the EPA’s UVR monitoring network was programmed to measure UV spectra during daylight hours (generally between 6 a.m. and 6 p.m. local time). A Brewer’s schedule for scanning the local sky was programmed based on zenith angle of the sun at its particular location. The first UV spectrum scan recorded each day by a Brewer occurred at a local solar zenith angle (SZA) of 85 degrees (i.e., approximately 5 degrees above the local horizon at sunrise). The instrument is programmed to make subsequent spectral scans at 5-degree increments after that point, since the SZA is changing rapidly. Near local noon, when the local SZA does not change rapidly, the scanning schedule was programmed to occur at intervals of approximately 20 min. On a typical summer day at mid-latitudes, approximately 30 UV spectral scans were recorded. The raw UV spectral data from each Brewer in the network was collected daily and processed through a data correction algorithm, which performed numerous quality control checks. The algorithm corrected the data for known systematic biases caused by temporal drift in Brewer calibration, non-ideal cosine response, and temperature dependence.

### 2.3. Determining Cloudiness in a Consistent Manner

To determine the dependence of the RAF on cloudiness, the parameter “cloudiness” must first be defined. Most Brewer sites had no additional instrumentation to independently measure clouds or broadband solar radiation, but general sky conditions were manually recorded in site operator logs. Therefore, Brewer UV spectral measurements were used to derive the cloudiness, or percent “clearness” (e.g., percent clear sky) indicator, where the sum of percent clearness and cloudiness equals 1.0. The selected indicator of percent clearness was based on the amount of light received with clouds compared to the amount of light received when the sky is cloudless. The percent clearness indicator was selected to be (1) independent of total column ozone, and (2) able to be determined as closely to the biologically damaging UV radiation measurement temporally as possible. Furthermore, the percent clearness indicator needed to be a value derived entirely from the recorded UV spectral data, rather than from the output of a radiative transfer model. The measurement of UV spectral irradiance is not an instantaneous measurement. The Brewer records UV spectral irradiance, progressing from shorter to longer wavelengths, while its internal diffraction grating is physically rotated. An entire UV spectral scan, from 286.5 nm to 363 nm, takes approximately six min. This yields a rate of approximately 13 nm/min of wavelength scan progression.

Although Brewers are capable of measuring total column ozone, the Brewer instruments in the EPA’s network were never initialized or calibrated to measure total column ozone. For that reason, NASA’s Total Ozone Mapping Spectrometer (TOMS) satellite provided total column ozone, in Dobson Units (DUs), from its daily overpass for each of the 10 sites analyzed in this study. Since only one daily satellite overpass was accomplished per site, the recorded daily total column ozone values were constant for each site. The biologically damaging UV radiation is largely determined from energy in wavelengths less than 320 nm (which are very sensitive to changes in total column ozone). The effective UV wavelength band range for ozone impacts occurs at wavelengths from 290 nm to 325 nm [19]. Changes in UVR flux at wavelengths of 320 nm and greater are associated with trends in aerosols, haze, and clouds, therefore wavelengths between 325 nm and 330 nm were selected to account for clouds as described below. There is less UVR sensitivity to ozone in this wavelength range.

**Figure 3:**
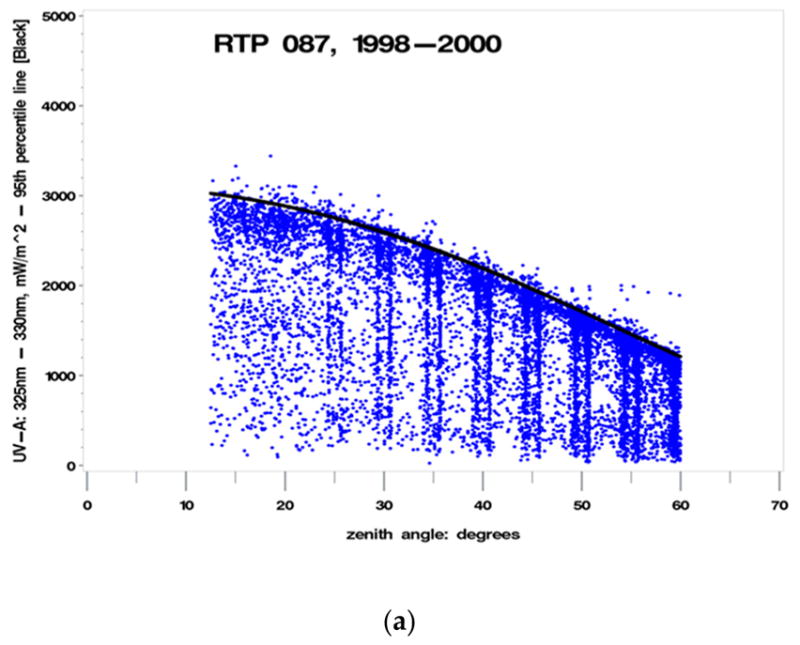

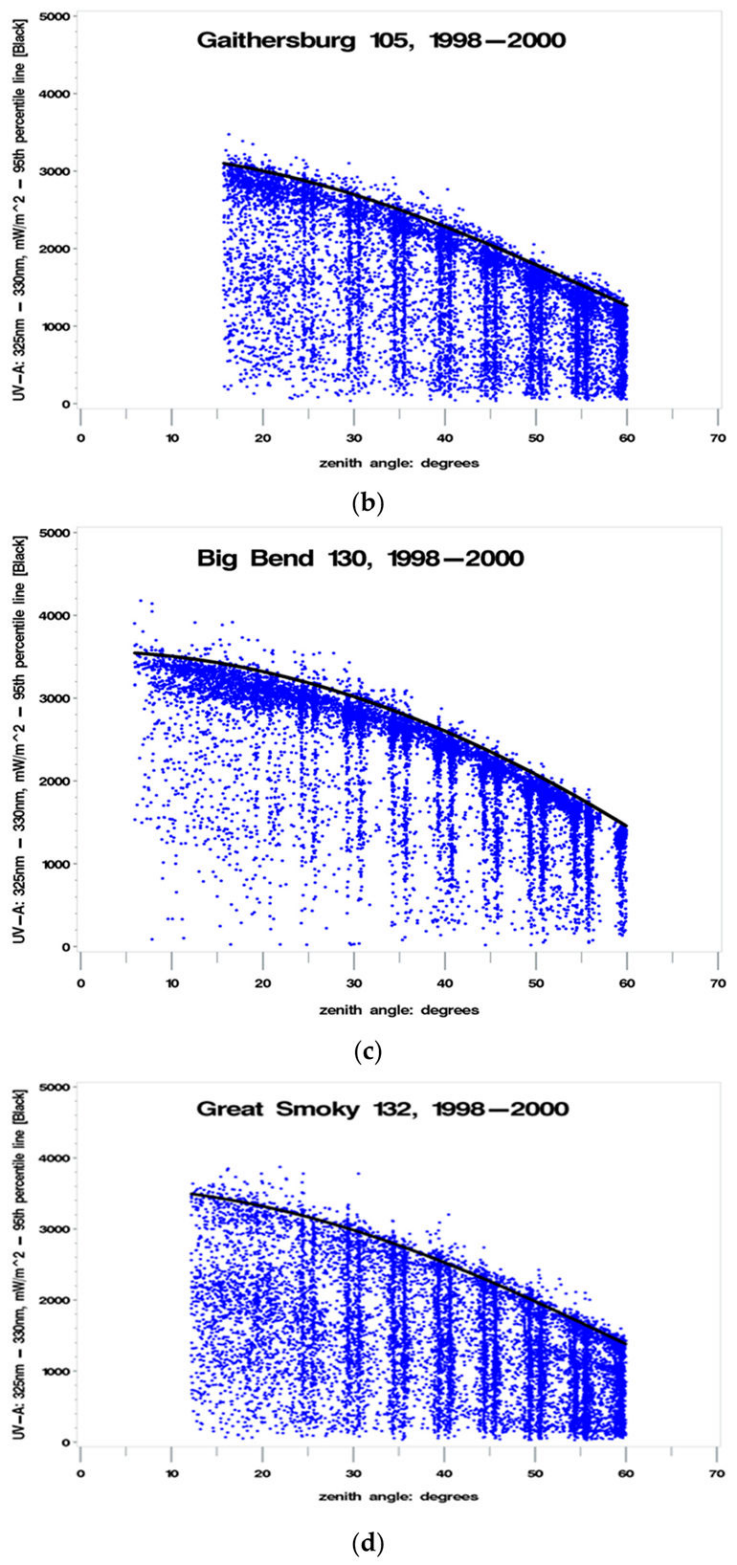

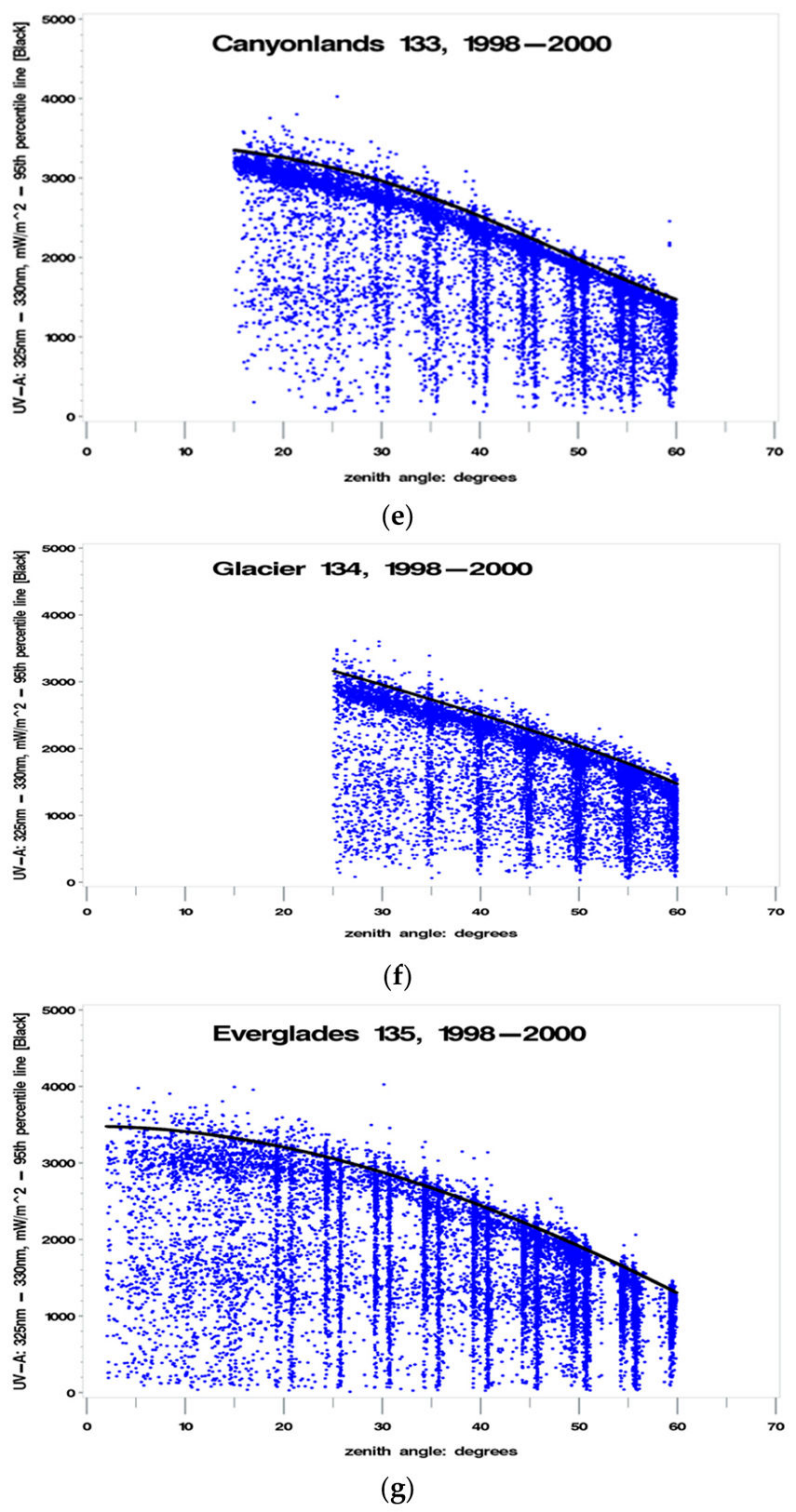

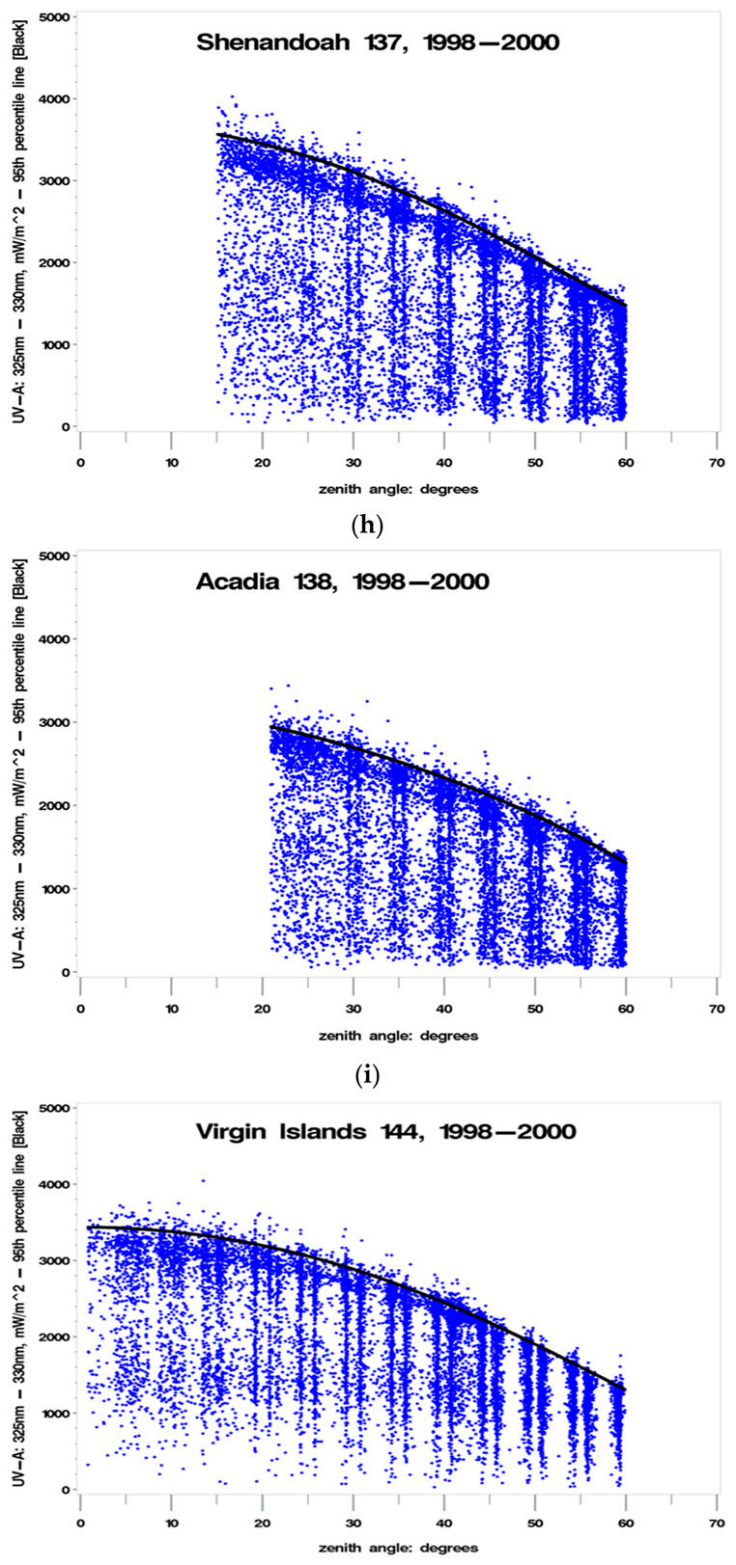
Plot of UV325 versus solar zenith angle (SZA) at (**a**) Research Triangle Park, NC; (**b**) Gaithersburg, MD; (**c**) Big Bend National Park, TX; (**d**) Great Smoky Mountain National Park, TN; (**e**) Canyonlands National Park, UT; (**f**) Glacier National Park, MT; (**g**) Everglades National Park, FL; (**h**) Shenandoah National Park, VA; (**i**) Acadia National Park, ME; (**j**) Virgin Islands National Park (St. John), USVI (CLR1, illustrated by the black line, represents the polynomial of the 95th percentile measurement at each SZA).

The UV spectral data scanned and recorded by the Brewers between 325–330 nm was used as the indicator for percent clearness. This particular wavelength segment was chosen because it lies above the wavelengths that are affected by total column ozone. The rational for selecting this wavelength segment (325–330 nm) was to ensure that any changes observed could be attributed to clouds and not to any potential impacts from total column ozone. This portion of the electromagnetic spectrum also provides a stable measurement basis. This wavelength segment is temporally close (within two min) to the wavelength segment which provides the major contribution to biologically damaging UV radiation. Given that the peak biologically damaging UV radiation contribution occurs at approximately 310 nm, the following calculation illustrates the temporal resolution (difference) between the wavelengths unaffected by total column ozone and those contributing to maximum biologically damaging UV radiation (e.g., the difference between 330 nm and 310 nm is 20 nm; with an instrument scan rate of 13 nm/min: 20 nm/(13 nm/min) = 1.538 min, yielding approximately 1.5 minutes’ temporal difference). The wavelength segment between 330–363 nm was also used, which is minimally affected by clouds, to ensure that a consistent definition of cloudiness could be determined at each site using the appropriate UV wavelength segment least impacted by clouds based on local site conditions. The rational for selecting this particular wavelength segment (330–363 nm) was to attribute observed changes to total column ozone and to remove impacts from clouds. For each of the 10 selected sites, a data set was generated with each observation consisting of four pieces of data including: (a) SZA measurement; (b) a total column ozone (O_3_) measurement from TOMS; (c) a biologically damaging UV radiation (Brewer) measurement; (d) an unweighted, integrated Brewer UVR measurement taken between the 325 nm and 330 nm wavelength segment (designated UV325). Using the data collected at the 10 selected UVR monitoring sites from 1998 through 2000, a plot of UV325 (in mW/m^2^) versus SZA, shown in Figure 3, clearly indicates an upper envelope of UV325, which is indicative of the percent clearness of the sky at a given SZA. The objective is to define separate indicators for percent (sky) clearness that are unaffected by total column ozone and unaffected by clouds.

Examination of the UV325 versus SZA plots for the 10 selected sites led to the determination of CLR1 (a parameter describing a cloud free day, based on the maximum range of UV irradiance values) which was added to the datasets, containing (a) through (d) above, from an SZA-dependent polynomial approximation of the 95th percentile of the UV325 measurements for each site {as shown by the black lines in Figure 3}. The polynomial regression fit for the 95th percentile is: UV_95_ = {cos(SZA)}^3^ + {cos(SZA)}^2^ + {cos(SZA)}, (0). The parameter CLR1 is a line which represents a boundary indicating where most (95%) of the UV measurements between 325 nm and 330 nm are found based on SZA at each particular site. CLR1 is the parameter describing a cloud free day, according to the maximum range of UV irradiance values, which would not occur on a cloudy or partially cloudy day. CLR1 tends to approach the upper limit of UV325 values at each SZA, but some values can exceed CLR1 values plotted along the 95th percentile line by up to 20 percent, most likely due to cloud reflections. The generated data set for each site could now be supplemented with an additional parameter (CLR1) derived from the UV325 versus SZA plots: CLR1 = UV325/(95th percentile of measurements at each SZA). The ten sites selected for the analysis are provided in Table 1. Brewer 105 was operated, maintained, and calibrated by the National Institute of Standards and Technology (NIST). Brewer 087 was operated and maintained by the US EPA. The remaining eight sites were operated, maintained, and calibrated by the NPS. The graphs, residuals, and histograms were generated using SAS^™^ statistical software.

The vertical lines shown in Figure 3 are an artifact of how the Brewer is configured to capture UV scan measurement data.

### 2.4. Variability in Biologically Damaging UV Radiation Data

It is preferable for conditions to remain constant during a scan, but that was not always the case. The temporal gap between the bulk of the contribution to the biologically damaging UV (at 310 nm) and the UV325 wavelength segment introduced some variability in the data. The variability appeared to be systematic, and can be illustrated through the following two examples. Hypothetical Situation: A day with widely scattered cumulus clouds; Example 1: At one point during the day, the sun is exposed (i.e., no clouds) at the beginning of an instrument spectral scan. Then, at the 320 nm wavelength portion of a spectral scan, a cloud passes in front of the sun. The calculated UV325 will be lowered to approximately 50% of the previous CLR1 value, and the measured biologically damaging UV radiation value will be higher than the UV325 value. Example 2: At another point during the same day, the sun is hidden in the early stages of a spectral scan and then emerges at approximately 320 nm, the UV325 will record nearly 100% clear, and will be higher than the previous CLR1 value, and the measured biologically damaging UV radiation value will be lower than the UV325 value. The frequency of such systematic variations tends to diminish as the sky becomes either heavily overcast (0% clear) or cloud-free (100% clear).

### 2.5. Relationship between Biologically Damaging UV Radiation, Cloudiness, Column Ozone, and RAF

**Figure 4:**
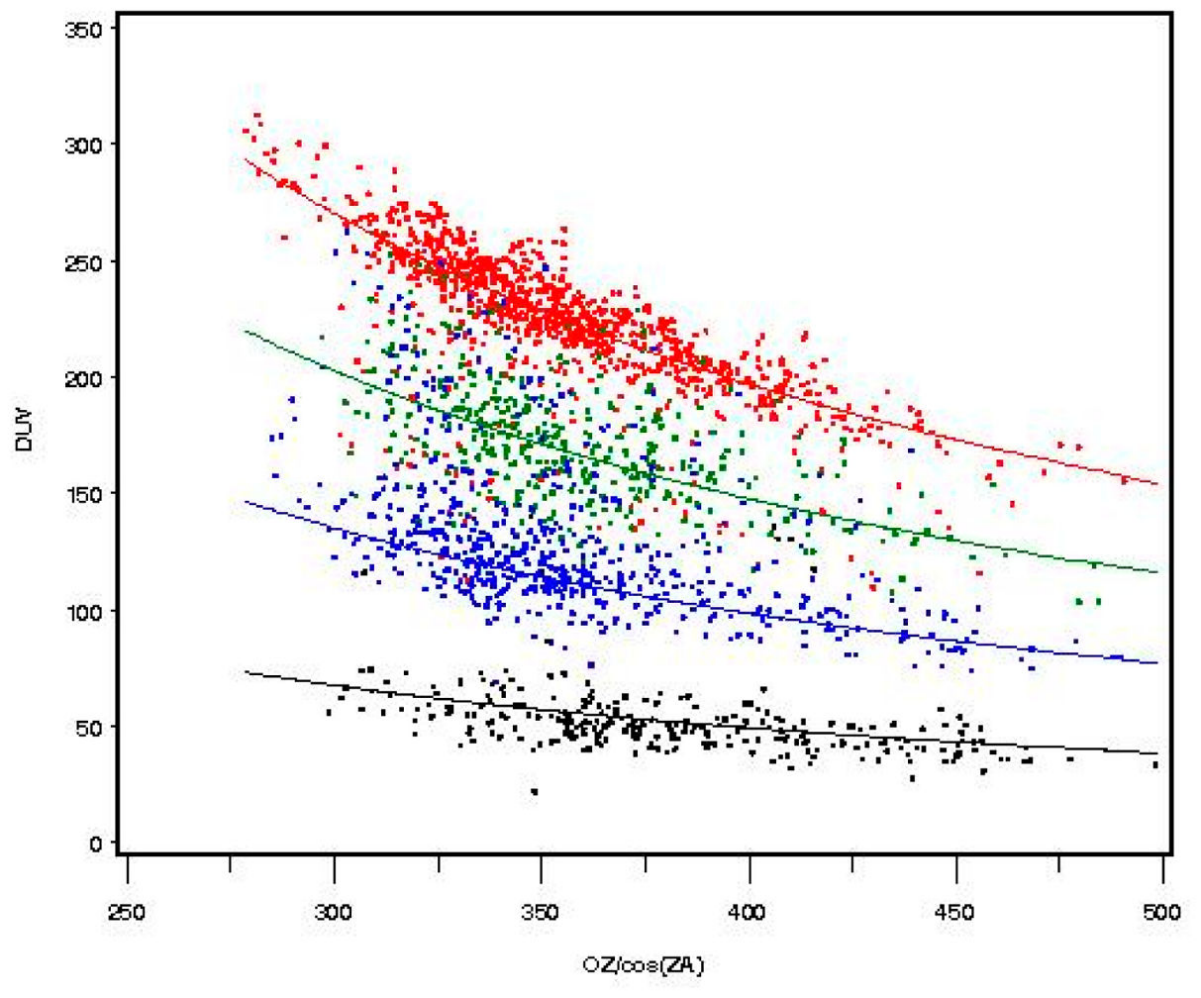
The biologically damaging UV radiation versus O_3_/cos(SZA) for percent clearness of 25%, 50%, 75%, and 100% (±3%) and SZA from 25 to 30 degrees. 100% (red) light clouds; 75% (green) thin clouds; 50% (blue) moderate clouds; 25% (black) thick clouds.

Data sets were compiled for the ten selected sites in the UVR network containing the measured parameters (SZA, total column ozone [O_3_], biologically damaging UV radiation in the erythemal action spectrum, UV325), and the derived CLR1 parameter. These parameters were then used to plot the biologically damaging UV radiation in the erythemal action spectrum versus total column ozone (O_3_), for different values of CLR1 and SZA. The biologically damaging UV radiation values were then normalized for the seasonal change in Earth–sun distance. Finally, although the polynomials defining “clear” (sky) UVR are approximately the same, there should be some systematic differences based on altitude, topographic obstruction, and haze/pollution at each site. For this analysis, the biologically damaging UV radiation values were normalized between sites to produce a nearly identical definition of “clear” sky. The data from all ten sites were then combined to produce the four plots shown in Figure 4 below, which display the biologically damaging UV radiation in the erythemal action spectrum versus total column ozone (O_3_) for percent clearness PCLR of 25% (black), 50% (blue), 75% (green), and 100% (red) ±3% and SZA from 25 to 30 degrees. The curved line relationship for the RAF = −1.1 is based on the power law:

(1) $$\text{Biologically}\;\text{Damaging}\;\text{UV}\;\text{Radiation}=\text{PCLR}\times A\times {\left((O_{3})/\cos\;(\text{SZA})\right)}^{-1.1}$$

where A is determined from the mean value of the biologically damaging UV radiation for total column ozone (O_3_), approximately 350 Dobson Units (DU).

There was considerable skewing of the measurements for the different percentages of clearness (100% clear [red, downward]) and (50% clear [blue, upward]) as would be expected from sudden appearance or disappearance of direct sun due to cloud configuration/movement, as discussed in two hypothetical examples described above. This skewing affected the regression analyses designed to determine the RAF theoretically.

Figure 4 illustrates that as total column ozone (O_3_) increases, biologically damaging UV radiation in the erythemal action spectrum decreases in a consistent manner. Clouds may have a systematic effect in altering the generally accepted value of a 1.1% decrease in the biologically damaging UV radiation in the erythemal action spectrum (–1.1%) for each 1.0% increase in total column ozone, as measured in Dobson Units (DUs).

### 2.6. Experimental Approach Used to Determine Radiation Amplification Factor (RAF)

An initial description of the relationship between cloud coverage and its effect on the RAF was obtained from 71,000 total UV measurement scans taken between 1998 and 2000 at ten Brewer sites in the EPA’s UVR Monitoring Network. The range of solar zenith angles for which measurements were taken was less than 60 degrees (60°). The relationship between the biologically damaging UV radiation, total column ozone (O_3_), cloudiness/percent clearness of the sky, and solar zenith angle was determined using the experimental setup. The RAF is a parameter which is designed to answer the question, “If total column ozone increases by one percent, what is the percentage change in the biologically damaging UV radiation (weighted integral of short wave UVR)”? If the assumption is that the RAF is a reasonable concept, an experiment must be designed to determine if there is a relationship between the RAF and cloudiness. The idea is to isolate the changes due to atmospheric ozone and minimize the impact of clouds when measuring changes in UVR at different wavelengths.

### 2.7. Ideal Experiment

An ideal experiment to assess the RAF would collect biologically damaging UV radiation measurements for a period of time and would be designed as follows. For a series of fixed SZA’s (e.g., constrained between SZA = 90 degrees [the local horizon at sunrise], with SZA = 0 being directly overhead, and SZA = −90 degrees [the local horizon at sunset]):

- Measure the total column ozone [O_3_] levels (using either TOMS satellite measurements or ground measurements with spectrophotometers as applicable, etc.) at each fixed SZA.
- Measure the biologically damaging UV radiation levels (using ground measurements with spectrophotometers, etc.) at each fixed SZA at measured:
  - Percent clearness = 0% [100% cloud cover] to Percent clearness = 100% [0% cloud cover]
  - Percent clearness = 100% to 120% [accounting for cloud “reflections”]—Note: enhancement of UVR by cloud reflections has been measured at up to 30 percent [20], therefore using a 20% enhancement (120%) for maximum biologically damaging UV radiation values provides a reasonable and conservative estimate where the percent clearness (cloudiness) would be measured by satellites and/or from ground-based instruments. Total column ozone was not measured using instruments at the sites, and cloudiness could not be assessed independent of human observation. The instruments could not (and cannot) measure biologically damaging UV radiation at fixed SZAs for extended periods, therefore the ideal experimental setup had to be adjusted to conform to the limitations of real-world constraints. When developing a preliminary methodology for implementing this hypothesis, an examination of the initial set of UV325 versus SZA plots that were generated by the statistical software indicated that “clear” was best defined as a SZA-dependent polynomial approximation of the 95th percentile of the UV325 measurements. This polynomial approximation was determined to be satisfactory, since it captured almost all of the upper values, and allowed some values to exceed clear by at least 20 percent, possibly due to cloud reflections, as noted in the literature [20].

### 2.8. Realizable Experiment (Driven by Real-World Constraints)

The total column ozone [O_3_] was measured, in DUs, from the TOMS satellite. The biologically damaging UV radiation is measured, in mW/m^2^, from the ground using Brewers. The computerized Brewer scan schedule does not accommodate measuring the biologically damaging UV radiation at fixed SZAs for long periods of time. There were no independent or reliable measurements or estimates of cloud cover either from ground-based measurements or from satellites at either Brewer site. However, operator logs at each site recorded the general sky condition and amount of cloudiness (e.g., overcast, rain, clear, sunny, etc.). This left the question of how to determine percent clearness (cloudiness) unanswered. Since there was no instrument-based measurement of cloudiness (percent clearness) available at either of the Brewer sites, a theoretically determined methodology for assessing percent clearness (cloudiness) was used as described below.

### 2.9. A Theoretical Definition of Cloudiness (Percent Clearness)

We used the wavelength segment between 325–330 nm (UV325) because it is minimally affected by total column ozone, and the wavelength segment between 330–363 nm (UV330) which is minimally affected by clouds. This was done so that a consistent and site-appropriate definition of cloudiness was used based on local site conditions, due to the differences in location, geography, climate, altitude, and ecology for each site. The polynomial representing the 95th percentile of all UVA measurements, between 330–363 nm, as a function of SZA is defined as 100 percent clear sky. The parameter CLR2, denoting the percentage of clear sky is calculated by:

(2) CLR2=(100×(UVA/95thpercentileofUVA))

### 2.10. Measured and Calculated Parameters

The parameters used in determining the RAF for each of the five modeling approaches, as displayed in Figures 6, 7, 13, 16, and 17, are as follows:

SZA = measured solar zenith angle of the sun, where SZA = 90 degrees [the local horizon at sunrise], SZA = 0 is directly overhead, and SZA = −90 degrees [the local horizon at sunset].
O_3_ = total column ozone (in Dobson Units)—from TOMS satellite measurements.
Biologically Damaging UV Radiation = Brewer spectrophotometer measurements.
CLR2 = percent clear (percent clear sky measurement at a selected SZA)—calculated value, derived from the polynomial representing the 95th percentile of all UVA measurements, between 330–363 nm, as a function of SZA.
To ensure that all potential confounding parameters are eliminated from the calculation and only RAF versus percent clear curves are generated for each modeling approach, the following steps were taken:
  - The UVR calculations were normalized with respect to the distance between the sun and the Earth during the year. Note: Earth is closest to sun on 5 January. The formula used to calculate the normalized UVR values is:
    (3) UVR={1-0.034cos(2π(jday-5)/365))}×BiologicallyDamagingUVRadiation
    where jday = 1, 2, …, 365, with jday = 5 being 5 January, [21].
  - The UVR values were normalized with respect to systematic differences due to site altitude, topography, pollution, aerosol, haze, etc.
  - The data from all ten sites was combined into curves of RAF versus percent clear sky for each of the five modeling approaches, Model A through Model E, (as shown in Figures 6, 7, 13, 16, and 17 respectively).

**Figure 5:**
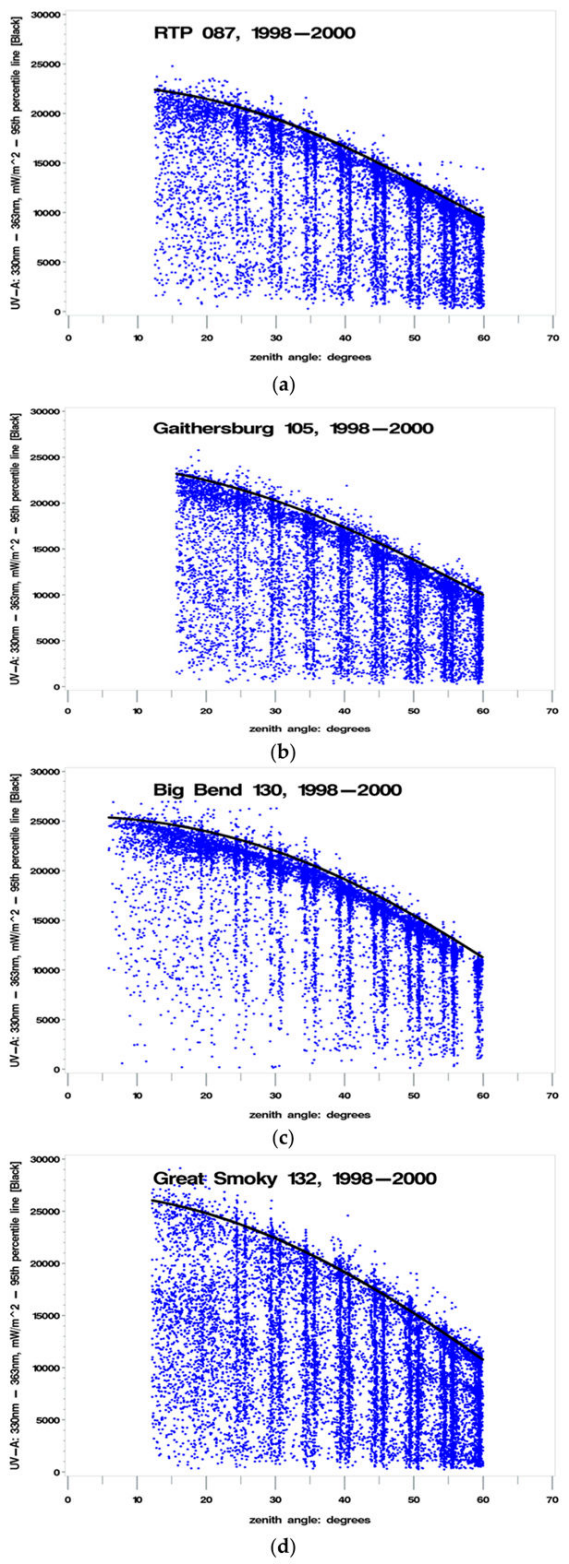

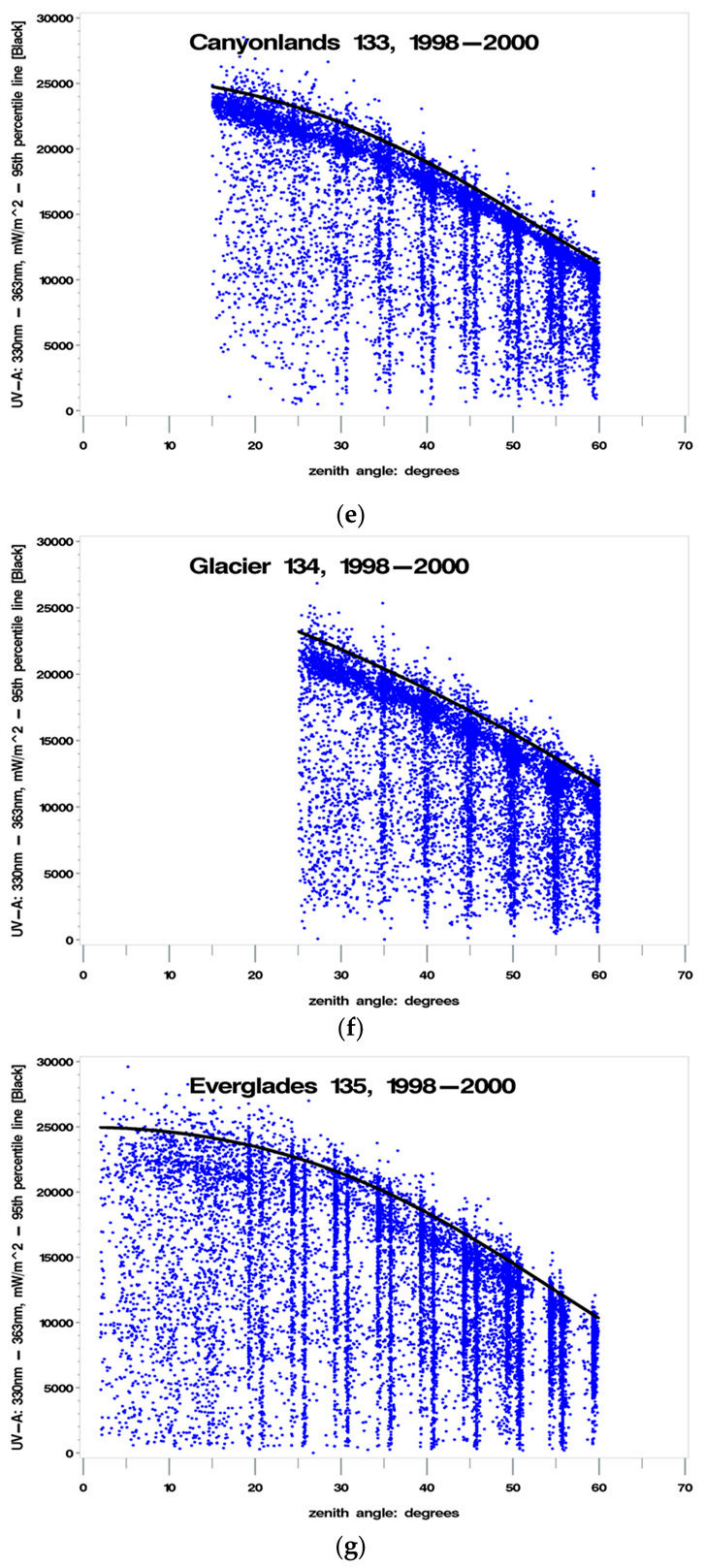

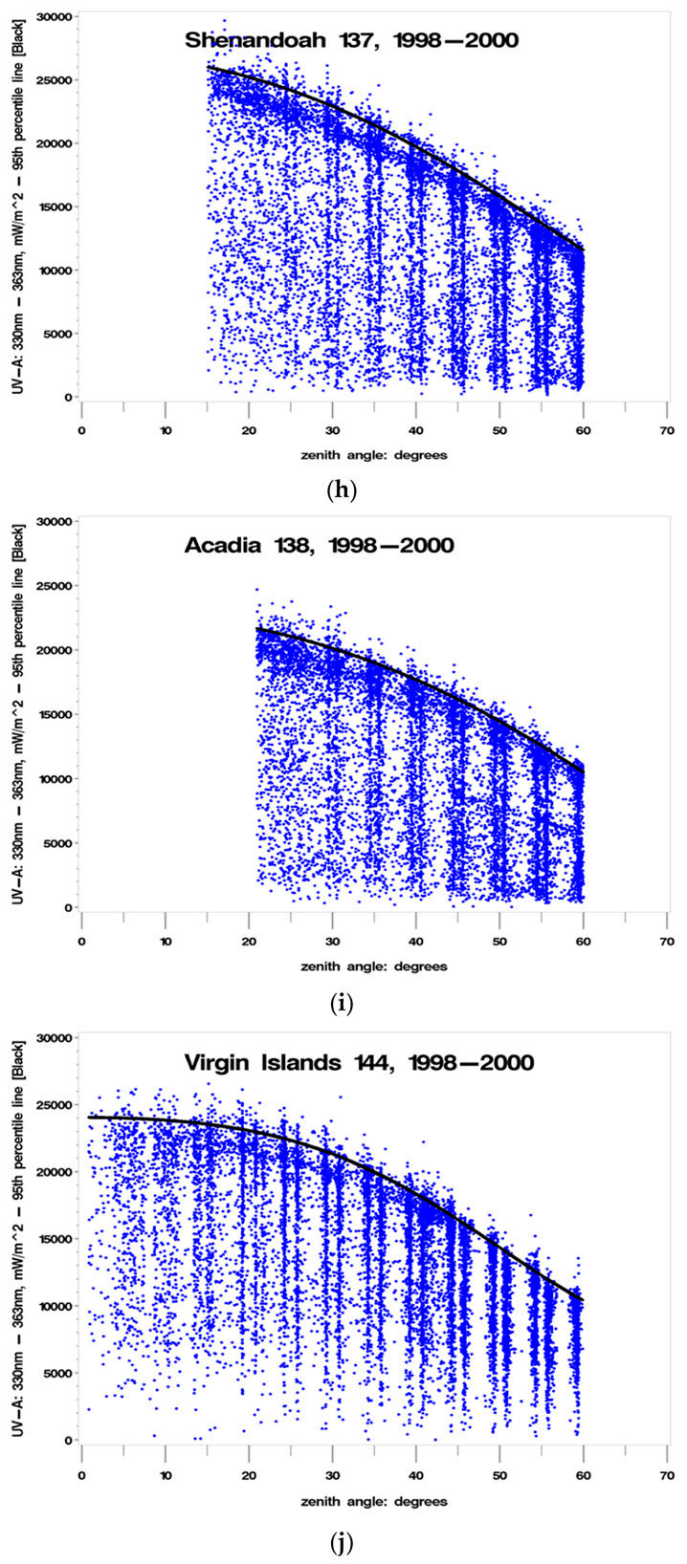
Plot of UV330 versus SZA at (**a**) Research Triangle Park, NC; (**b**) Gaithersburg, MD; (**c**) Big Bend National Park, TX; (**d**) Great Smoky Mountain National Park, TN; (**e**) Canyonlands National Park, UT; (**f**) Glacier National Park, MT; (**g**) Everglades National Park, FL; (**h**) Shenandoah National Park, VA; (**i**) Acadia National Park, ME; (**j**) Virgin Islands National Park (St. John), USVI (CLR2, illustrated by the black line, represents the polynomial of the 95th percentile measurement at each SZA).

Figure 5 illustrates the unweighted UVR measurements taken between the 330–363 nm wavelength segments. This wavelength segment is minimally affected by clouds, and is designated as UV330. The 95th percentile envelope for UV330, defined by a non-linear polynomial approximation for each site, is identical in shape to that of UV325, matching the behavior of CLR1 in that wavelength segment over the same SZA range, with the only difference being that the power values are higher for the UV330 envelopes.

For each of the 10 selected sites, a data set was generated with each observation consisting of four pieces of data including: (a) solar zenith angle (SZA) measurement; (b) a total column ozone (O_3_) measurement from TOMS; (c) biologically damaging UV radiation (Brewer measurement); (d) an unweighted, integrated Brewer UVR measurement taken between the 330 nm and 363 nm wavelength segment (designated UV330). Using the data collected at the 10 selected UVR monitoring sites from 1998 through 2000, a plot of UV330 (in mW/m^2^) versus SZA, shown in Figure 5, clearly indicates an upper envelope of UV330, which is indicative of the percent clearness of the sky at a given SZA.

Examination of the UV330 versus SZA plots for the 10 selected sites led to the determination of the derived “clear” (CLR2) parameter added to the datasets, (containing a through d above), from an SZA-dependent polynomial approximation of the 95th percentile of the UV330 measurements for each site {as shown in Figure 5}. CLR2 tends to approach the upper limit of UV330 values at each SZA, but some values can exceed CLR2 values plotted along the 95th percentile line by up to 20 percent, most likely due to cloud reflections. The generated data set for each site could now be supplemented with an additional parameter (CLR2) derived from the UV330 versus SZA plots: CLR2 = UV330/(95th percentile of measurements at each SZA).

The vertical lines shown in Figure 5 are an artifact of how the Brewer is configured to capture UV scan measurement data. During the analysis of the UVR data, various parameters were kept in a limited range such as SZA, at less than 60 degrees (60°), and wavelengths, at less than 330 nm, to ensure that only the effect of clouds would manifest itself in the analysis. There were five modeling approaches used in the analysis of the 71,000 data points analyzed between 1998 and 2000 at the 10 UVR monitoring sites. Four of the five modeling approaches are based on “thought experiments”, using empirical data, and one model is based on a purely empirical relationship (Beer–Lambert’s Law). The five different modeling approaches were compared to each other, and these five models each related the biologically damaging UV radiation to the following set of parameters (as illustrated in Table 2: Model A—(1) total column ozone [O_3_], (2) percent (%) clear sky {i.e., percent clearness/cloudiness} [CLR], (3) SZA; Model B—(1) total column ozone [O_3_], (2) “clear” [replaces the “CLR” term, and is calculated by performing a regression on percentage “classes” of cloud cover, and the result of the regression is called iclear]; “iclear” is the parameter describing cloudiness (as discrete percentage levels grouped into different classes), based on the maximum range of UV irradiance values, (3) SZA; Model C—(1) total column ozone [O_3_], (2) “iclear*” [replaces the “CLR” term, and is calculated by performing a regression on percentage “classes” of cloud cover, with the removal of the tails of the biologically damaging UV radiation distribution, i.e., censor “bad” data (remove the extreme values below the 2.5th percentile and above the 97.5th percentile), and the result is called iclear*], (3) SZA—Note: This is the same as Model B, except with the tails of the biologically damaging UV radiation distribution removed; Model D—(1) total column ozone [O_3_], (2) CLR^^^ [replaces CLR with a linear function in percent clearness {“classes” of CLR} between 40% and 100% clear skies], (3) SZA; Model E—(1) total column ozone [O_3_]/cos(SZA), and (2) cos(SZA): This is an empirical relationship known as Beer–Lambert’s Law. At the 10 UVR monitoring sites selected for the analysis, the NASA Total Ozone Mapping Spectrometer (TOMS) satellites were used to measure total column ozone. This provided the benefit of an independent measurement source for determining atmospheric (total column) ozone.

## 3. Results

Each model is based on the 71,000 total UVR measurements taken at the ten sites listed in Table 1 between 1998 and 2000. The five modeling approaches, Model A through Model E, all yield average RAF values of approximately −1.1 for all sky conditions (e.g., 0% to 120% clear sky) at the ten selected UVR monitoring sites. A consistent “definition” of sky cloudiness (clearness) was provided at each site using the segment of UVR wavelengths least impacted by clouds (or ozone as applicable). This provided a stable baseline for comparison of each set of model-derived RAF values. Since Model E was solely based on an empirical relationship, it can be compared against the four hypothetical models (A through D). The summary results for each modeling approach is described below.

Model A: The average RAF value from Model A for all 10 sites and for all sky conditions (i.e., in % [sky] clearness from 0% to 120%: parameter—CLR) and solar zenith angles less than 60° is −1.167. Model A is the base model from which Models B, C, and D are derived.
Model B: The average RAF value for all 10 sites and for all sky conditions and solar zenith angles less than 60° is −1.1936. This model performed a regression on the discrete “classes” of the parameter CLR and replaced it with the result (called iclear). This model produced the highest average RAF value of the five models.
Model C: The average RAF value for all 10 sites and for all sky conditions (i.e., in % [sky] clearness from 0% to 120%: parameter—iclear*) and solar zenith angles less than 60° is −1.105. This model is identical to Model B, except data censoring was applied (i.e., the “tails” of the biologically damaging UV radiation distribution [below the 2.5th percentile and above the 97.5th percentile] was removed).
Model D: The average RAF for all 10 sites and for all sky conditions (i.e., in % [sky] clearness from 0% to 120%: parameter—CLR^^^) and solar zenith angles less than 60° is −1.087. The parameter representing the percentage of clear sky (CLR^^^), was assumed to be a linear function between 40% and 100% clear skies. This model provided the lowest average RAF value of the five models.
Model E: The average RAF value for all 10 sites and for all sky conditions (i.e., in % [sky] clearness from 0% to 120%) and solar zenith angles less than 60° is −1.119. Model E is based on a physical/empirical model (Beer–Lambert’s Law).

The overall average RAF value from the five methods used for the ten selected network sites was −1.134, with the RAF values for individual methods ranging from a low of −0.80 to a high of −1.38, as shown in Table 2. The ensemble average of the low RAF values resulting from the five models, applied across the ten selected sites, is −0.864. The ensemble average of the high RAF values resulting from the five models, applied across the ten selected sites, is −1.302. The comparison of the five models is presented in Table 2.

### 3.1. Experimental Determination of the RAF: Five Modeling Approaches

#### 3.1.1. Model A

The relationship between the biologically damaging UV radiation and the following parameters: (1) total column ozone [O_3_]; (2) CLR [percent (%) clear sky {i.e., percent clearness/cloudiness}]; (3) solar zenith angle [SZA], is given as follows (where a = RAF, and b and c are regression coefficients):

(4) $$\text{Biologically}\;\text{Damaging}\;\text{UV}\;\text{Radiation}=A\times {\left(O_{3}\right)}^{a}\times {\left(\text{CLR}\right)}^{b}\times {\left(\cos(\text{SZA})\right)}^{c}$$

The slope of the UVR–ozone relationships derived for clear skies, light cloudy skies, medium cloudy skies and heavy cloudy skies for this model, can be found using the formula:

(5) $$\ln\;(\text{Biologically}\;\text{Damaging}\;\text{UV}\;\text{Radiation})=\ln\;(A)+a\times \text{ln}\;(O_{3})+b\times \ln\;(\text{CLR})+c\times \ln\;(\cos(\text{SZA}))$$

(with ln representing the natural logarithm) which can be rewritten as:

(6) $$\ln\;(\text{Biologically}\;\text{Damaging}\;\text{UV}\;\text{Radiation})=a\times \ln\;(O_{3})+b\times \ln\;(\text{CLR})+c\times \ln\;(\cos(\text{SZA}))+d$$

where d = ln (A), a constant.

**Figure 6:**
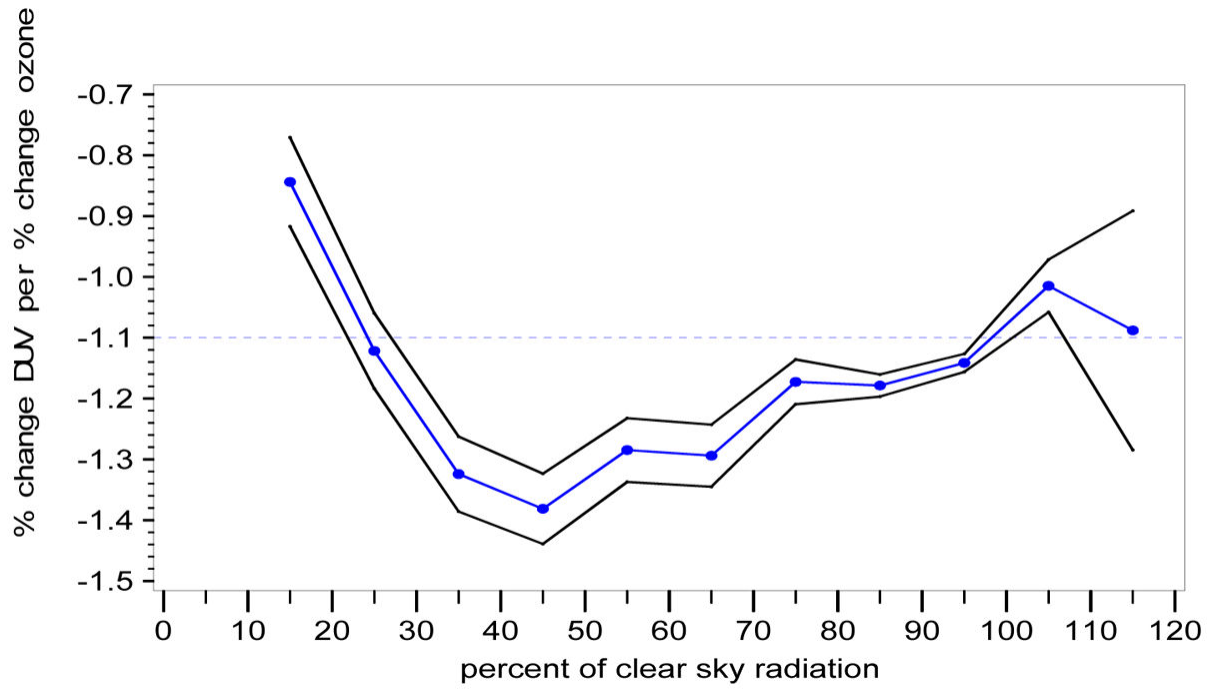
Model A: RAF versus percent clear sky radiation transmitted (cloudiness) parameter (CLR).

If we generate a graph of “a”, where a = RAF, as a function of CLR (% clearness, where 100% clear is defined as the 95th percentile of all integrated UVR measurements for 330–363 nm [where cloud effects are minimized], as a function of SZA = solar zenith angle), the black line envelope in Figure 6 gives the 2-sigma (95%) confidence interval around the value of the RAF.

With Model A, the RAF tends to increase (i.e., become less negative) as the clear sky percentage decreases (from 45% clear sky radiation [transmitted] to 15% clear sky radiation [transmitted]). This behavior is expected, as an increase in cloudiness (decrease in percent clear sky radiation transmitted) tends to reduce the amount of UV radiation reaching the Earth’s surface for each 1% change in total column ozone. At 45% clear sky radiation, where the lowest RAF value (−1.38) is found, there is an inflection point, after which RAF values tend to increase in general, with negligible decreases (between 55% to 65% clear sky radiation and between 105% to 115% clear sky radiation).

#### 3.1.2. Model B

The relationship between the biologically damaging UV radiation and the following parameters: (1) total column ozone [O_3_]; (2) “iclear” [Note: to obtain “iclear”, replace the “CLR” term {used in Model A}, with a regression performed on “classes” of CLR; the result of the regression on the “classes” of CLR is called “iclear”]; (3) solar zenith angle [SZA], is given as follows:

(7) $$\text{Biologically}\;\text{Damaging}\;\text{UV}\;\text{Radiation}=A\times {\left(O_{3}\right)}^{a}\times {\left(\text{CLR}\right)}^{b}\times {\left(\cos(\text{SZA})\right)}^{c}$$

The slope of the UVR–ozone relationships derived for clear skies, light cloudy skies, medium cloudy skies and heavy cloudy skies for this model, can be found using the formula used in Model A:

(8) $$\ln\;(\text{Biologically}\;\text{Damaging}\;\text{UV}\;\text{Radiation})=\ln\;(A)+a\times \ln\;(O_{3})+b\times \ln\;(\text{CLR})+c\times \ln\;(\cos(\text{SZA}))$$

which can be rewritten in its derivative form as:

(9) $$[\delta \text{Biologically}\;\text{Damaging}\;\text{UV}\;\text{Radiation}/\delta O_{3}]/\text{Biologically}\;\text{Damaging}\;\text{UV}\;\text{Radiation}=a/(O_{3})+b\times [\delta \text{CLR}/\delta O_{3}]/\text{CLR}$$

Note: The ln (A) term and the c × ln (cos(SZA)) term both are reduced to 0 when derivatives are taken since ln (A) is a constant and cos(SZA) is a constant, since 0 < cos(SZA) < 1. Equation (9) can be reduced to:

(10) $$[\Delta \text{Biologically}\;\text{Damaging}\;\text{UV}\;\text{Radiation}/\text{Biologically}\;\text{Damaging}\;\text{UV Radiation}]/[\Delta O_{3}/O_{3}]=a=\text{RAF}$$

**Figure 7:**
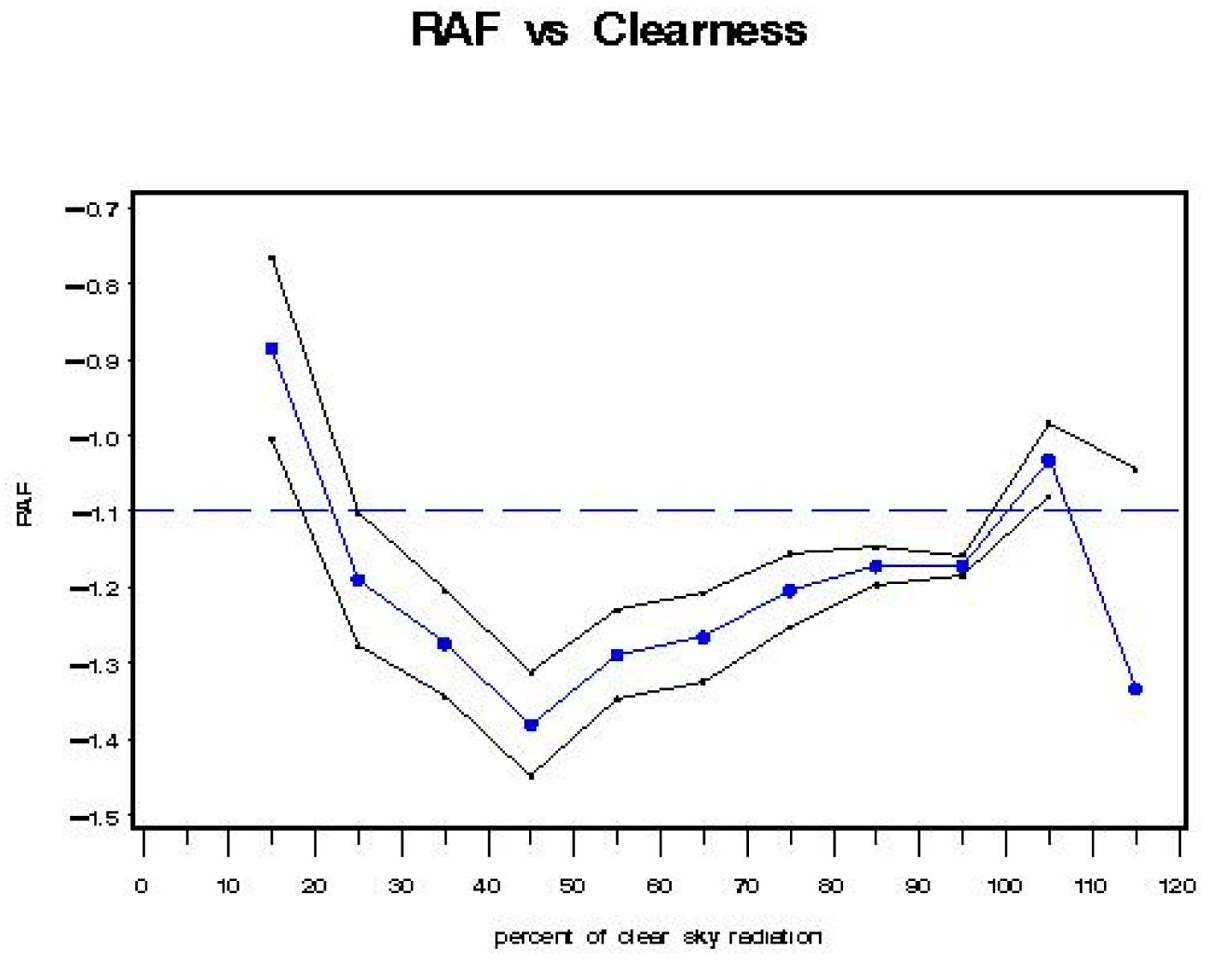
Model B: RAF versus percent clear sky radiation transmitted (cloudiness) parameter using “classes” of percent clear sky radiation transmitted (variable: iclear).

If we generate a graph of “a”, where a = RAF, as a function of the regression on each class of CLR (iclear—where, for example, for CLR representing 40–50% clear skies, iclear = 45; for CLR representing 90–100% clear skies, iclear = 95%, etc.), the black line envelope in Figure 7 gives the 2-sigma (95%) confidence interval around the value of the RAF.

With Model B, the RAF tends to increase (i.e., become less negative) as the clear sky percentage decreases (from 45% clear sky radiation [transmitted] to 15% clear sky radiation [transmitted]). This behavior is expected, as an increase in cloudiness (decrease in percent clear sky radiation transmitted) tends to reduce the amount of UV radiation reaching the Earth’s surface for each 1% change in total column ozone. At 45% clear sky radiation, where the lowest RAF value (−1.375) is found, there is an inflection point, after which RAF values tend to increase in general, with a constant region between 85% to 95% clear sky radiation and a decreasing region between 105% to 115% clear sky radiation.

**Figure 8:**
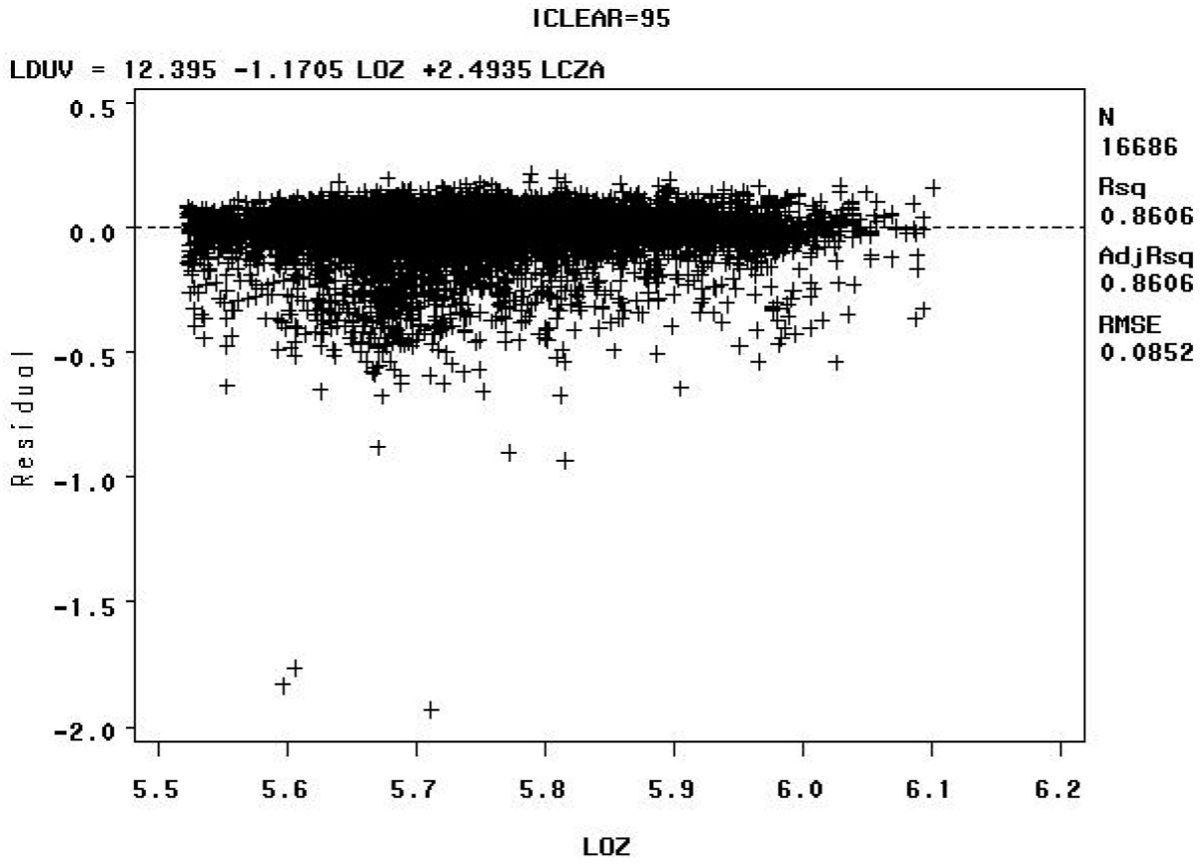
Model B: Residuals versus the natural logarithm of the total column ozone value in Dobson Units (LOZ) for sky clearness class 90% to 100% (iclear = 95)—Note: LCZA = natural logarithm of the cosine of the solar zenith angle, LDUV = natural logarithm of the biologically damaging UV radiation, and LOZ = natural logarithm of the total column ozone.

The Model B residuals versus the natural logarithm of the total column ozone value in Dobson Units (LOZ), for clear sky radiation between 90–100% (e.g., iclear = 95), may indicate some biologically damaging UV radiation well below the expected values as shown in Figure 8 below.

**Figure 9:**
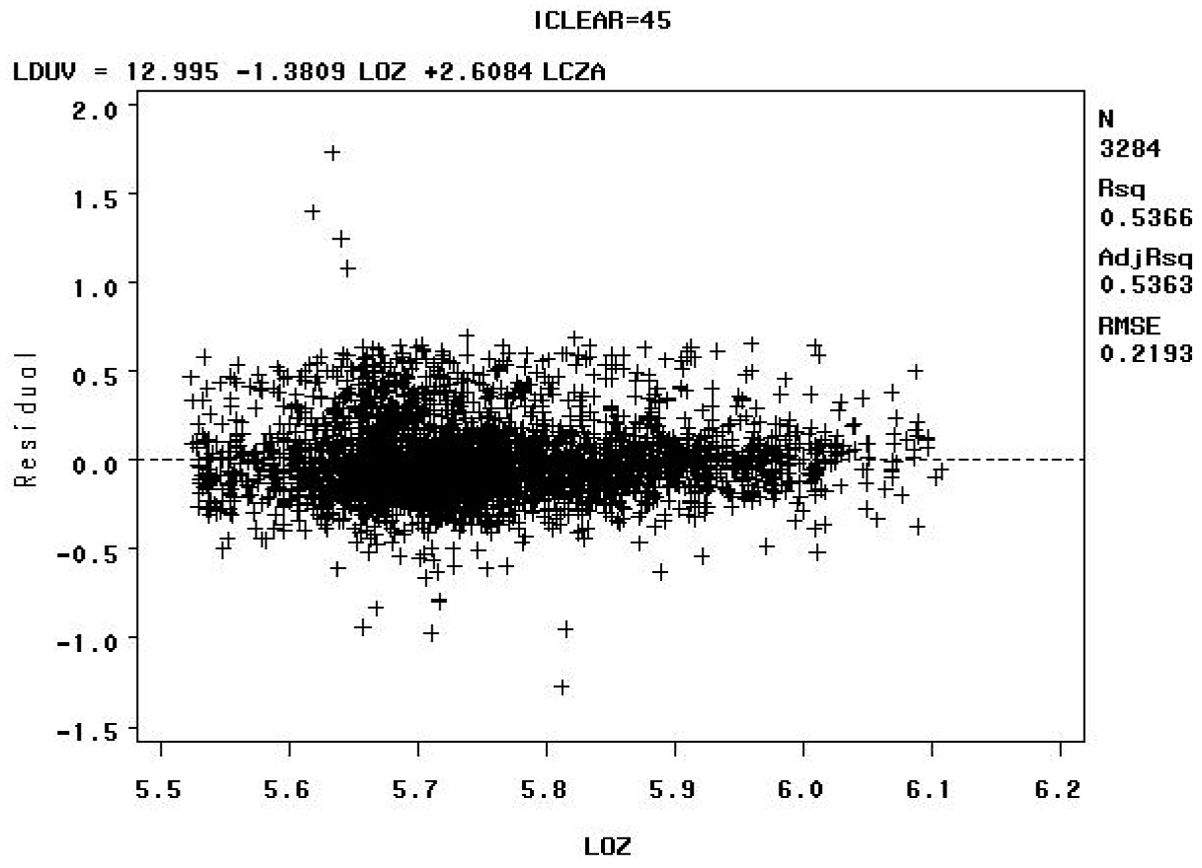
Model B: Residuals versus the natural logarithm of the total column ozone value in Dobson Units (LOZ) for sky clearness class 40% to 50% (iclear = 45)—Note: LCZA = natural logarithm of the cosine of the solar zenith angle, LDUV = natural logarithm of the biologically damaging UV radiation, and LOZ = natural logarithm of the total column ozone.

The Model B residuals versus the natural logarithm of the total column ozone value in Dobson Units (LOZ) for sky clearness percentages of 40–50% (iclear = 45) may indicate some biologically damaging UV radiation well above the expected values, as shown in Figure 9 below.

The residuals for the for sky clearness class 90% to 100% (iclear = 95), Figure 8, and for the sky clearness class 40% to 50% (iclear = 45), Figure 9, would seem to indicate that Model B fits the data well. But note that residuals vs. LOZ for iclear = 95 seems to indicate some biologically damaging UV radiation well below the expected values. The residuals vs. LOZ for iclear = 45 may indicate some biologically damaging UV radiation well above the expected values.

**Figure 10:**
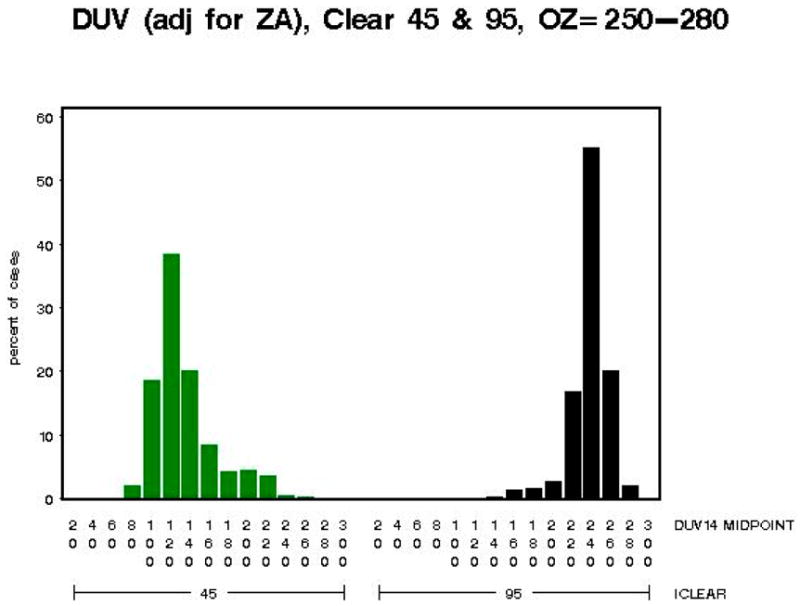
Model B: The biologically damaging UV radiation (adjusted for SZA) for 45% clear skies (green) and 95% clear skies (black) for total column ozone from 250 Dobson Units (DU) to 280 DU—Note: *x*-axis shows distribution of biologically damaging UV radiation wavelengths seen at 45% clear skies (green) and 95% clear skies (black) and the y-axis shows the percentage of biologically damaging UV radiation at each wavelength range.

The distributions of the biologically damaging UV radiation as shown in Figure 10 are skewed in opposite directions for iclear = 45 (right) and iclear = 95 (left). Partial cloud covering during the measurement of the spectrum (which takes six min to complete) is a potential cause of the skewed biologically damaging UV radiation distributions.

**Figure 11:**
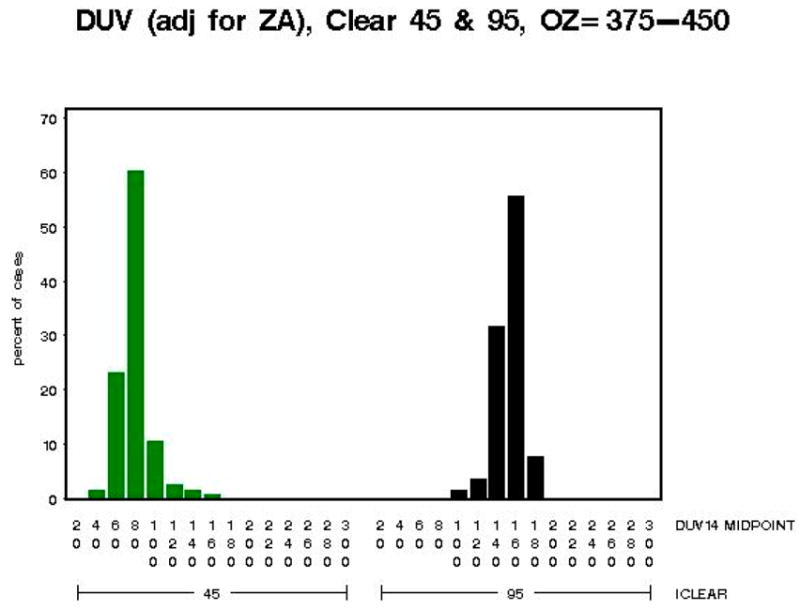
Model B: The biologically damaging UV radiation (adjusted for SZA) for 45% clear skies (green) and 95% clear skies (black) for total column ozone from 375 DU to 450 DU—Note: *x*-axis shows distribution of biologically damaging UV radiation wavelengths seen at 45% clear skies (green) and 95% clear skies (black) and the *y*-axis shows the percentage of biologically damaging UV radiation at each wavelength range.

The distributions of the biologically damaging UV radiation as shown in Figure 11 for iclear = 45 and iclear = 95 are less skewed, and closer to normality, for this larger total column ozone range as compared to the total column ozone range (250–280 DU) in Figure 10.

**Figure 12:**
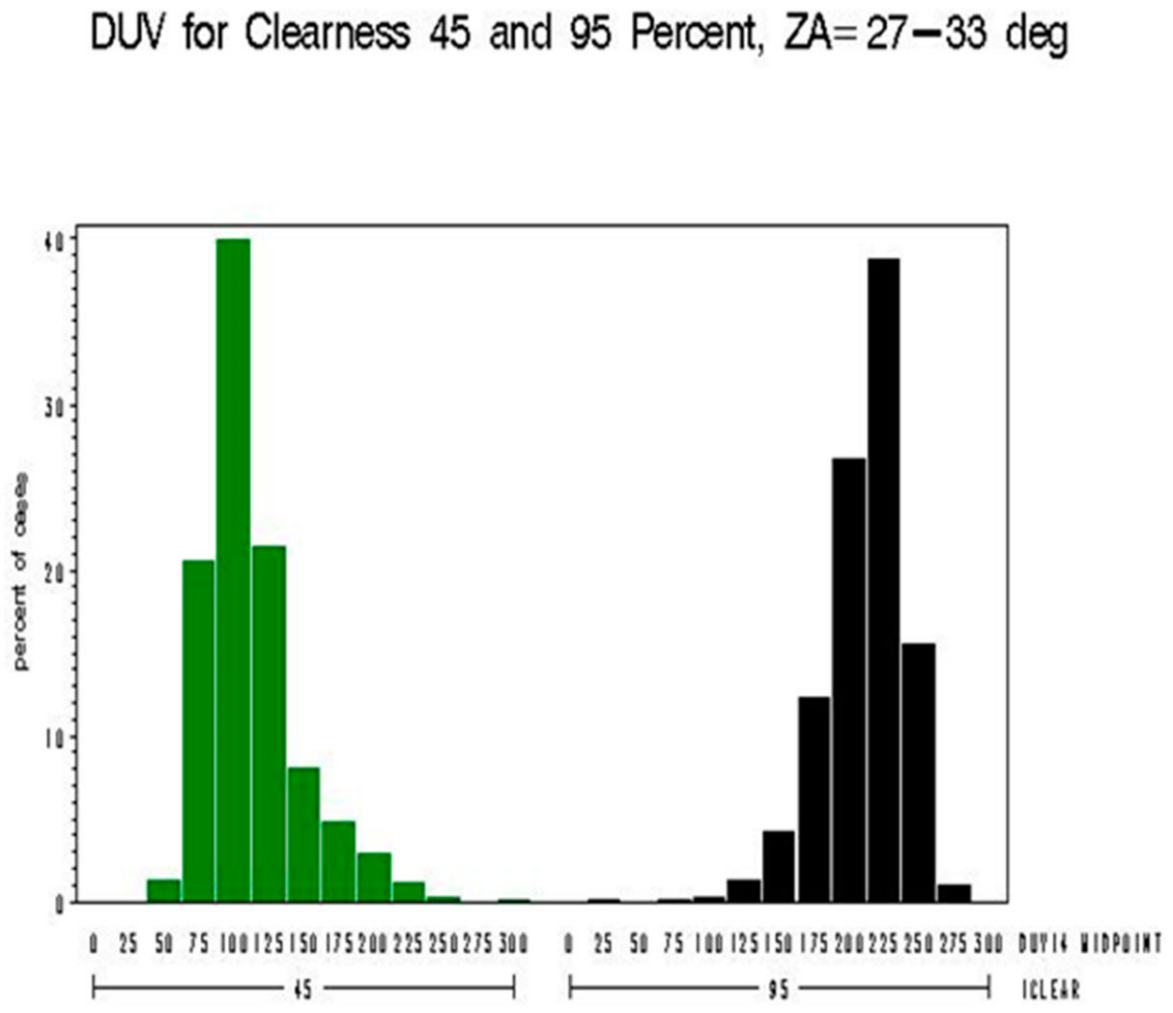
Model B: The biologically damaging UV radiation for 45% clear skies (green) and 95% clear skies (black) for solar zenith angles from 27 degrees to 33 degrees—Note: *x*-axis shows distribution of biologically damaging UV radiation wavelengths seen at 45% clear skies (green) and 95% clear skies (black) and the *y*-axis shows the percentage of biologically damaging UV radiation at each wavelength range.

The distributions of the biologically damaging UV radiation as shown in Figure 12 are skewed in opposite directions for iclear = 45 (right) and iclear = 95 (left) for solar zenith angles constrained near 30 degrees.

#### 3.1.3. Model C

The relationship between the biologically damaging UV radiation and the following parameters: (1) total column ozone [O_3_]; (2) “iclear” [Note: to obtain “iclear”, replace the “CLR” term {used in Model A}, with a regression performed on “classes” of CLR; the result of the regression on the “classes” of CLR is called “iclear*”, and remove the tails of the biologically damaging UV radiation distribution, i.e., censor “bad” data; (3) solar zenith angle [SZA], is identical to Model B (using the same equations) except the tails of the biologically damaging UV radiation distributions are removed (data censoring).

**Figure 13:**
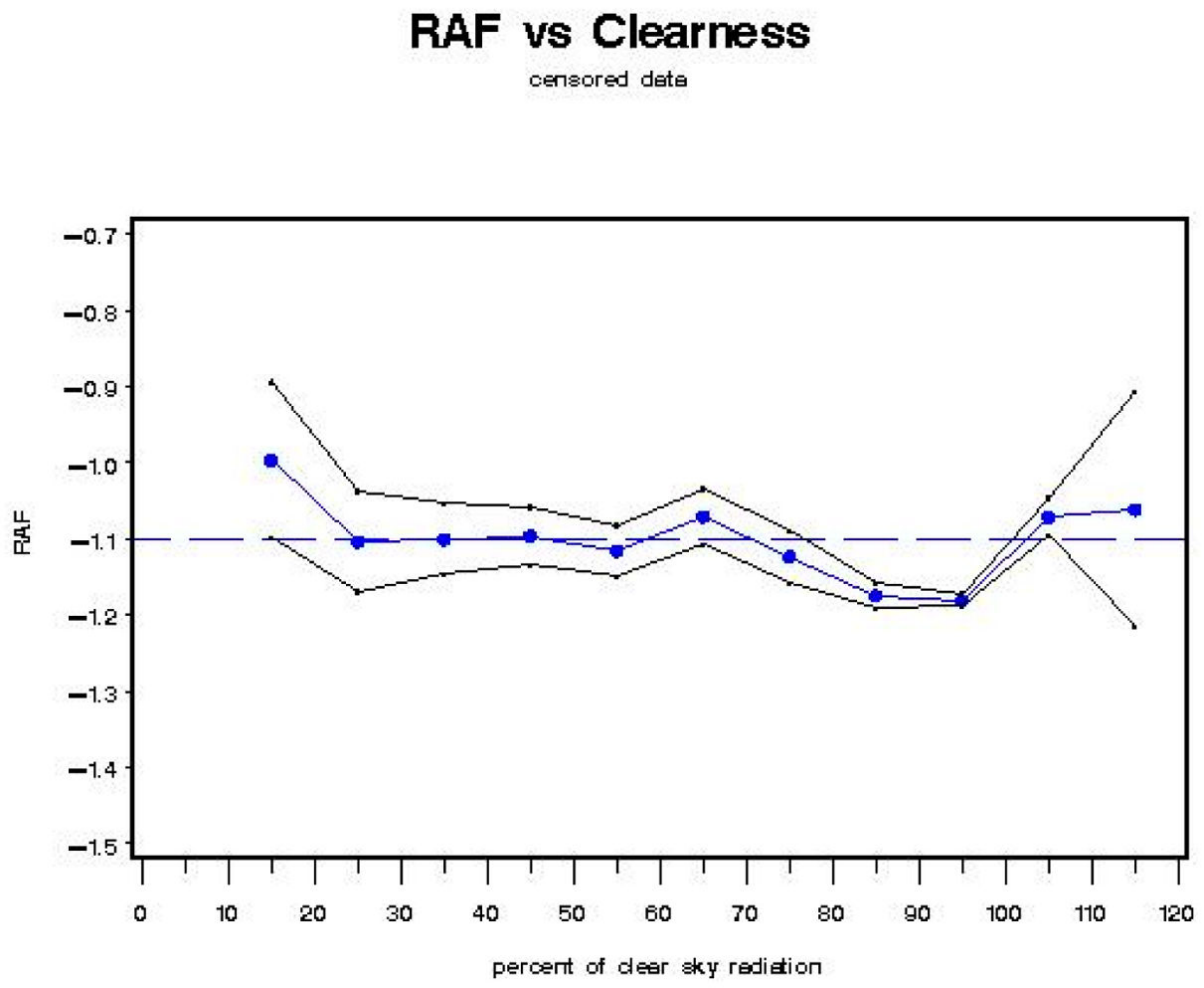
Model C: RAF versus percent clear sky radiation transmitted (cloudiness) parameter using “classes” of percent clear sky radiation transmitted (variable: iclear)—with removal of the “tails” of the biologically damaging UV radiation distributions.

If we generate a graph of “a”, where a = RAF, as a function of the regression on each class of CLR (iclear—where, for example, for CLR representing 40–50% clear skies, iclear = 45; for CLR representing 90–100% clear skies, iclear = 95%, etc.), with data censoring (biologically damaging UV radiation distribution tails removed), the black line envelope in Figure 13 gives the 2-sigma (95%) confidence interval around the value of the RAF. When the data in the tails of the biologically damaging UV radiation distributions are removed, there is minimal effect of percent clear sky (cloudiness) on the RAF as compared to Model A or Model B.

**Figure 14:**
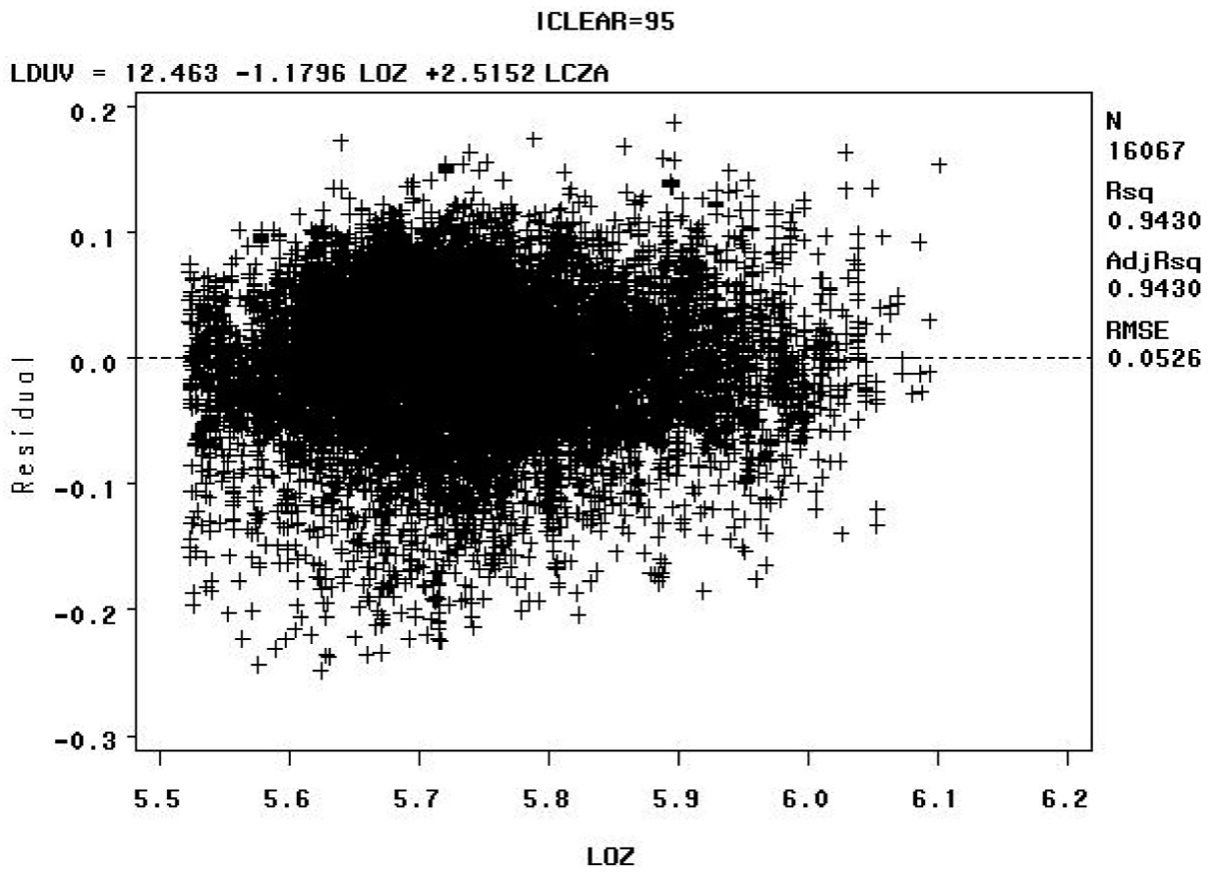
Model C: Residuals versus the natural logarithm of the total column ozone value in Dobson Units (LOZ) for sky clearness class 90% to 100% (iclear = 95) with removal of the “tails” of the biologically damaging UV radiation distributions—Note: LCZA = natural logarithm of the cosine of the solar zenith angle, LDUV = natural logarithm of the biologically damaging UV radiation, and LOZ = natural logarithm of the total column ozone.

**Figure 15:**
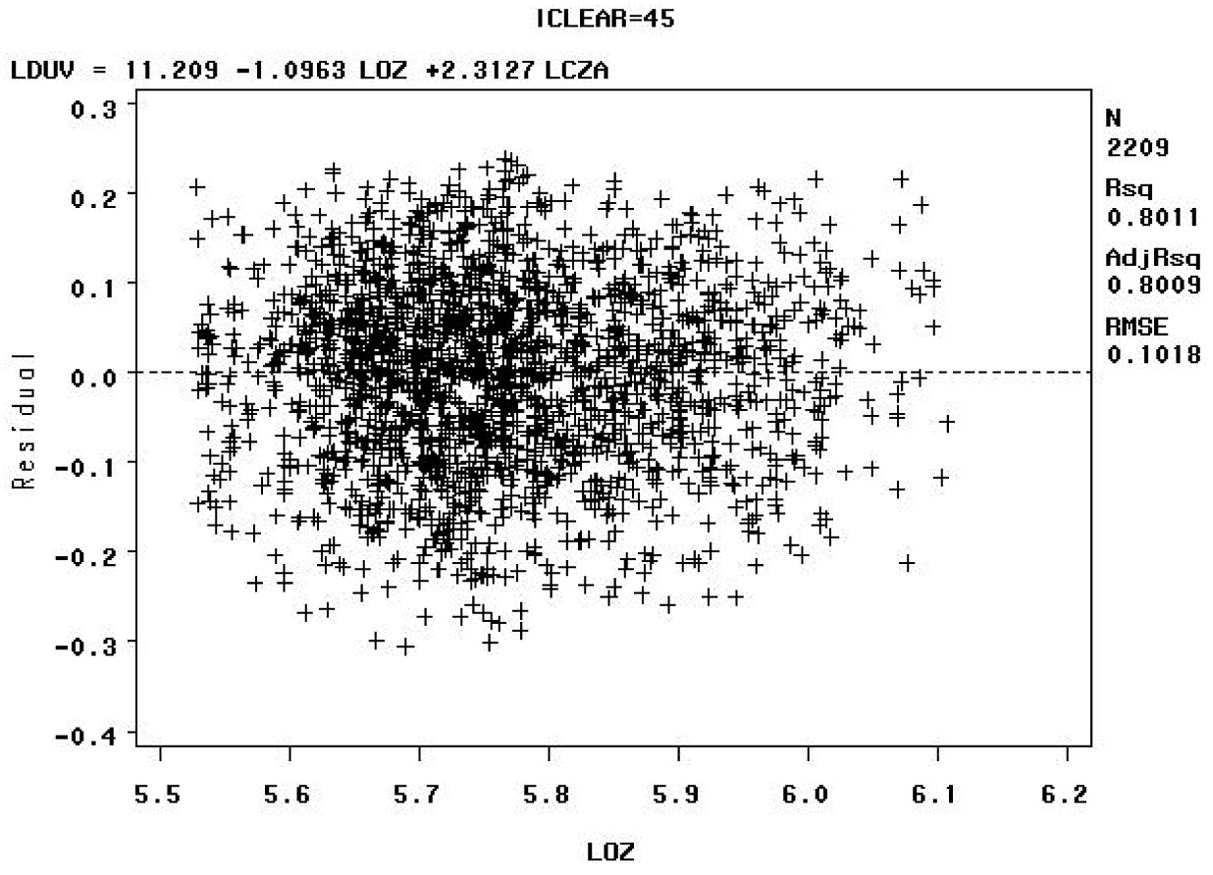
Model C: Residuals versus the natural logarithm of the total column ozone value in Dobson Units (LOZ) for sky clearness class 40% to 50% (iclear = 45) with removal of the “tails” of the biologically damaging UV radiation distributions—Note: LCZA = natural logarithm of the cosine of the solar zenith angle, LDUV = natural logarithm of the biologically damaging UV radiation, and LOZ = natural logarithm of the total column ozone.

With Model C, the RAF tends to increase (i.e., become less negative) as the clear sky percentage decreases (from 25% clear sky radiation [transmitted] to 15% clear sky radiation [transmitted]). This behavior is expected, as an increase in cloudiness (decrease in percent clear sky radiation transmitted) tends to reduce the amount of UV radiation reaching the Earth’s surface for each 1% change in total column ozone. Between 25% and 45% clear sky radiation, there is no visible change in RAF value. This may be a result of the removal of the “tails” of the biologically damaging UV radiation distributions, which would be expected to drive the values of the distributions closer to the mean value. This is the only model without an inflection point at 45% clear sky value, which seems reasonable given the censoring of the tails of the biologically damaging UV radiation distributions. The lowest RAF value (−1.18) occurs at 95% clear sky radiation, which again might be attributable to the data censoring used in this model. The residuals for Model C censored data are provided in Figures 14 and 15.

The Model C residuals versus the natural logarithm of the total column ozone value in Dobson Units (LOZ) for sky clearness percentages of 90–100% (iclear = 95) indicate that biologically damaging UV radiation mostly clusters around the expected values, with a high R^2^ value, as shown in Figure 14 below. The high R^2^ value for Model C can be attributed to the fact that extreme values were removed by data censoring. The R^2^ values for the residuals of the other models are lower than Model C and display more scatter.

The Model C residuals versus the natural logarithm of the total column ozone value in Dobson Units (LOZ) for sky clearness percentages of 40–50% (iclear = 45) indicate that these biologically damaging UV radiation measurements have a larger spread than those at sky clearness percentages of 90–100% (iclear = 95), with a relatively high R^2^ value, as shown in Figure 15 below.

At first glance, the residuals for the for sky clearness class 90% to 100% (iclear = 95), Figure 14, and for the sky clearness class 40% to 50% (iclear = 45), Figure 15, would seem to indicate that Model C fits the data well, but the fact that the tails of the distribution were removed does not allow there to be an accurate assessment of model fit by analyzing residuals.

#### 3.1.4. Model D

The relationship between the biologically damaging UV radiation and the following parameters: (1) total column ozone [O_3_]; (2) CLR^^^ [assuming a linear function in percent clearness {“classes” of CLR} for skies between 40% clear and 100% clear]; (3) solar zenith angle [SZA], is given as follows:

(11) BiologicallyDamagingUVRadiation=A×(O3)a+b×CLR^×(cos(SZA))c

where a + b × CLR^^^ represents a linear function in percent clearness for 40–100% clear skies.

The slope of the UVR–ozone relationships derived for clear skies, light cloudy skies, medium cloudy skies and heavy cloudy skies for this model, can be found using the formula:

(12) ln(BiologicallyDamagingUVRadiatoin)=ln(A)+a×ln(O3)+b×CLR^×ln(O3)+c×ln(cos(SZA))

which can be rewritten in its derivative form as:

(13) [δBiologicallyDamagingUVRadiation/δO3]/BiologicallyDamagingUVRadiation=(a+b×CLR^)/(O3)

Note: The ln (A) term and the c × ln (cos(SZA)) term both are reduced to 0 when derivatives are taken since ln (A) is a constant and cos(SZA) is a constant, since 0 < cos(SZA) < 1. Equation (13) can be reduced to:

(14) [ΔBiologicallyDamagingUVRadiation/BiologicallyDamagingUVRadiation][ΔO3/O3]=(a+b×CLR^)=RAF

**Figure 16:**
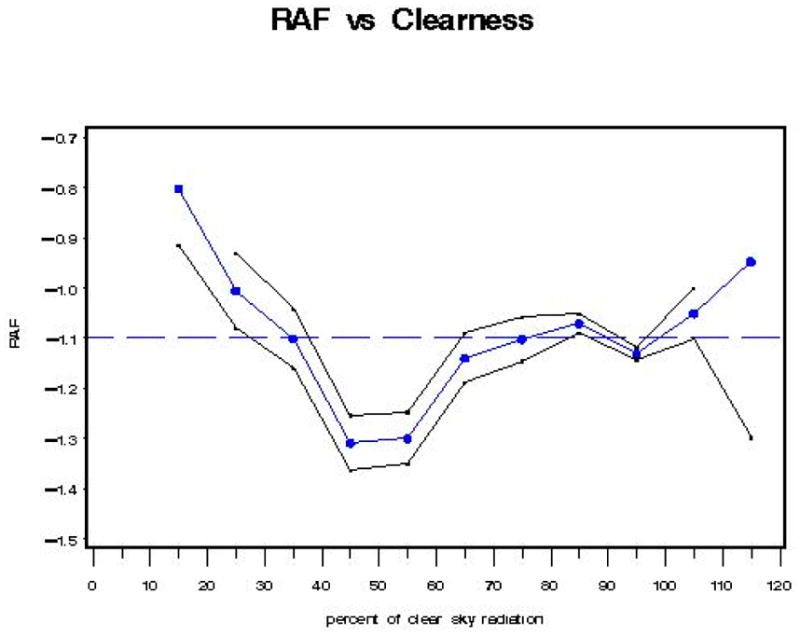
Model D: RAF versus percent clear sky radiation transmitted (cloudiness) parameter (CLR^^^): a linear function between 40% and 100% clear sky radiation transmitted.

The black line envelope in Figure 16 gives the 2-sigma (95%) confidence interval around the value of the RAF. The results were similar to those found in Model B for individual regressions using the “iclear*” parameter to represent classes of percent clearness. Note in Figure 16 the “dip” in RAF values between 45–55% clearness. This could indicate isolated clouds passing over the sun during individual measurement scan periods.

With Model D, the RAF tends to increase (i.e., become less negative) as the clear sky percentage decreases (from 45% clear sky radiation [transmitted] to 15% clear sky radiation [transmitted]). This behavior is expected, as an increase in cloudiness (decrease in percent clear sky radiation transmitted) tends to reduce the amount of UV radiation reaching the Earth’s surface for each 1% change in total column ozone. At 45% clear sky radiation, where the lowest RAF value (−1.3) is found, there is an inflection point, after which RAF values increase, with one exception between 85–95% clear sky radiation. The RAF values between 40–60% clear sky radiation could possibly indicate an isolated cloud over the sun during a scan.

#### 3.1.5. Model E—(A Model Based on an Empirical Relationship: Beer–Lambert’s Law)

The functional relationship between the biologically damaging UV radiation and the following parameters: total column ozone [O_3_]/cos(SZA), and cos(SZA), based on Beer–Lambert’s Law is shown in Equation (15) (where k = −RAF/{O_3_/cos(SZA)}):

(15) $$\text{Biologically}\;\text{Damaging}\;\text{UV}\;\text{Radiation}=A\times \exp\;(-k\times O_{3}/\cos(\text{SZA}))\times {\left(\cos(\text{SZA})\right)}^{c}$$

The slope of the UVR–ozone relationships derived for clear skies, light cloudy skies, medium cloudy skies and heavy cloudy skies for this model, can be found using Equation (16):

(16) $$\ln\;(\text{Biologically}\;\text{Damaging}\;\text{UV}\;\text{Radiation})=\ln\;(A)-k\times O_{3}/\cos(\text{SZA})+c\times \ln\;(\cos(\text{SZA}))$$

Which can be rewritten in its derivative form as:

(17) $$\left[\delta \text{Biologically}\;\text{Damaging}\;\text{UV}\;\text{Radiation}/\delta O_{3}]/\text{Biologically}\;\text{Damaging}\;\text{UV}\;\text{Radiation}=-k\times O_{3}/\cos(\text{SZA}\right)$$

Note: The ln (A) term and the c × ln (cos(SZA)) term both are reduced to 0 when derivatives are taken since ln (A) is a constant and cos (SZA) is a constant, since 0 < cos(SZA) < 1. Equation (17) can be reduced to:

(18) $$[\Delta \text{Biologically}\;\text{Damaging}\;\text{UV}\;\text{Radiation}/\text{Biologically}\;\text{Damaging}\;\text{UV}\;\text{Radiation}]/[\Delta O_{3}/O_{3}]=-k\times O_{3}/\cos(\text{SZA})=\text{RAF}$$

Using fixed values for average total column ozone and average solar zenith angle facilitates sensitivity analysis of the RAF.

(19) $$\text{RAF}=-k\times \text{avg}\;O_{3}/\cos(\text{avg}\;\text{SZA}):[\text{avg}\;O_{3}=315\;\text{DU},\;\text{avg}\;\text{SZA}=36\;\deg]$$

**Figure 17:**
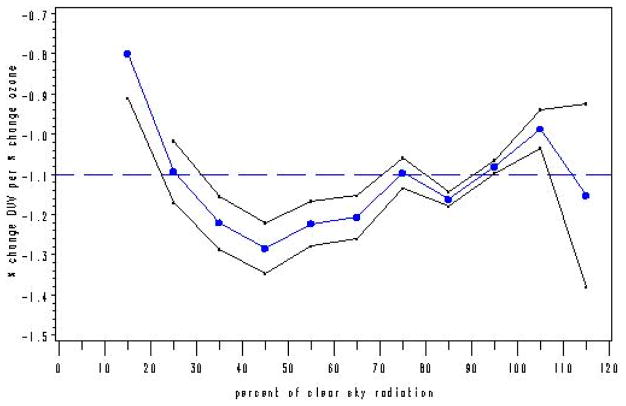
Model E: RAF versus percent clear sky radiation transmitted (cloudiness) parameter: {O_3_/cos(SZA)/cos(SZA)}.

In order to make the definition of UV-A temporally closer to the definition of the biologically damaging UV radiation, for this model (Model E), use the UVR wavelength range of 325–330 nm {UV325: Figure 3}, which is unaffected by ozone, not 330–363 nm (Figure 5) used previously in Models A through D. Since the empirical relationship is proportional to total column ozone, it is necessary to find the portion of the electromagnetic spectrum where UVR is unaffected by atmospheric ozone and observe the variation of SZA and cloudiness on biologically damaging UV radiation. The black line envelope in Figure 17 gives the 2-sigma (95%) confidence interval around the value of the RAF. Beer–Lambert’s Law suggests that a more suitable physical model implies that the RAF is not constant but is proportional to (O_3_/cos(SZA)). However, using average ozone and SZA values yielded results similar to those provided in Figure 17. Note in Figure 17 the “dip” in RAF values between 40–50% clearness. This could indicate isolated clouds passing over the sun during individual measurement scan periods. Note: There may be a relationship between the seasonal ozone cycle (i.e., high ozone = spring, low ozone = fall), cloud types, and cloud coverage amounts requiring further exploration.

With Model E, the RAF tends to increase (i.e., become less negative) as the clear sky percentage decreases (from 45% clear sky radiation [transmitted] to 15% clear sky radiation [transmitted]). This behavior is expected, as an increase in cloudiness (decrease in percent clear sky radiation transmitted) tends to reduce the amount of UV radiation reaching the Earth’s surface for each 1% change in total column ozone. At 45% clear sky radiation, where the lowest RAF value (−1.28) is found, there is an inflection point, after which RAF values increase, with exceptions between 75% and 85% clear sky radiation, and between 105% and 115% clear sky radiation, consistent with [22].

Although Model A and Model B have average RAF values closer to −1.2, while Model D has an average value of nearly −1.1 (−1.087), Model A, Model B, and Model D behave in a similar fashion, as they each display the relationship where RAF decreases as clear sky increases (percent cloudiness decreases) between the 100% to 120% clear sky values. Each of these three models displays the lowest RAF values (−1.3 to −1.38) at the 45% clear sky value. Due to the censoring of the tails of the biologically damaging UV radiation distribution, understandably, Model C has a “flattened” RAF versus clear sky response curve. Model E, like Model A, Model B, and Model D, also has its lowest RAF value (−1.28) at the 45% clear sky value. The average RAF value of Model E is −1.119 (less than Model A), and it displays the relationship where RAF increases as clear sky increases (percent cloudiness decreases) between the 85% to 105% clear sky values, unlike Models A, B, and D.

## 4. Conclusions

This research project utilized a large dataset of UVR measurements from ten monitoring sites that were diverse with respect to spatial location, geography, climate, altitude, and ecology to calculate RAF using four hypothetical approaches and one empirical model. This research project developed a consistent definition of cloudiness, independent of total column ozone, and temporally close to biologically damaging UV radiation measurements to facilitate direct and consistent comparisons between each of the approaches for modeling RAF. Model C, which censors the tails of the biologically damaging UV radiation distribution, should not be used to assess RAF, since the RAF value is artificially driven to a mean value (−1.1). While Models A, B, and D are consistent and comparable in the relationship between RAF and clear sky radiation between 100% and 120%, the empirically-based model, Model E, behaves differently in that range.

The results show that for all models except Model C (censored tails), in the 35% to 65% range of clear sky radiation (65% to 35% cloudiness), as cloudiness decreases, RAF increases, ranging from −1.2 to −1.4, where it reaches its highest level before decreasing. Between 15% and 25% clear sky radiation (85% to 75% cloudiness), there is an increase in RAF for all models. In general, RAF tends to increase as the sky conditions change from high to moderate cloudiness. Clouds block biologically damaging UV radiation in this range and the behavior is qualitatively the same as if stratospheric ozone was increased. UVR can experience a 4.3% increase per kilometer of altitude, from 0 km to 6.2 km, and is also affected by atmospheric pressure and aerosol density [23]. It is noted in the literature that mean cloud attenuation of UVR in the UV-B (wavelengths: 280–315 nm) is approximately 15–30% [24]. UV radiation is also reflected by both clouds and the Earth’s surface [25], which complicates analyses in this area. Erythemally-weighted UV-B has displayed a 3–7% decadal increase in mid and high latitudes from Nimbus 7 satellite observations [26]. For 105% to 120% clear sky radiation, RAF increases for Models A, B and E, while decreasing for Models C and D. The RAF is important because it serves as a sensitivity metric that directly relates changes in stratospheric ozone to changes in the UVR flux reaching the Earth’s surface. Overall, the resulting RAF values from the five modeling approaches are consistent with those found in [27].

## Figures and Tables

**Table 1:** Ten EPA UV Radiation Research Program Sites Used in the Analysis.

Brewer Number	Site Location	GPS Latitude	GPS Longitude	Elevation (Meters)	Elevation (Feet)	Start Date	Site Type
087	Research Triangle Park (RTP), NC	35.8924 N	78.8771 W	104	341	1995	Urban
105	Gaithersburg, MD	39.1342 N	77.2167 W	43	141	1994	Urban
130	Big Bend, TX	29.3050 N	103.1770 W	329	1079	1997	National Park
132	Great Smoky Mountains, TN	35.6044 N	83.7829 W	564	1850	1996	National Park
133	Canyonlands, UT	38.4584 N	109.8211 W	814	2671	1997	National Park
134	Glacier, MT	48.7409 N	113.4325 W	424	1391	1997	National Park
135	Everglades, FL	25.3906 N	80.6805 W	18	59	1997	National Park
137	Shenandoah, VA	38.5226 N	78.4349 W	325	1066	1997	National Park
138	Acadia, ME	44.3769 N	68.2609 W	137	449	1998	National Park
144	St. John, USVI	18.3360 N	64.7960 W	30	98	1998	National Park

**Table 2:** Comparison of the RAF Values for Modeling Approaches Used.

Model	Low RAF	High RAF	Functional Relationship	Calculate RAF—Use Functional Relationship between Biologically Damaging UV Radiation and:
A	−0.84	−1.38	biologically damaging UV radiation ~f (O_3_, CLR, SZA)	total column ozone [O_3_]
				CLR [percent (%) clear sky {i.e., percent clearness/cloudiness}]
				solar zenith angle [SZA]
B	−0.88	−1.375	biologically damaging UV radiation ~f (O_3_, iclear, SZA)	total column ozone [O_3_]
				“iclear” [eliminate CLR term, and perform a regression on “classes” of CLR, and replace the parameter CLR with the result of the regression on the “classes” of clear, called iclear]
				solar zenith angle [SZA]
C	−1.00	−1.18	biologically damaging UV radiation ~f (O_8_, iclear^#^, SZA)—with data censoring (tails of the biologically damaging UV radiation distribution)	total column ozone [O_3_]
				revised “iclear” [eliminate CLR term, and perform a regression on “classes” of CLR, and replace the parameter CLR with the result of the regression on the “classes” of clear, called iclear, and remove the tails of the biologically damaging UV radiation distribution, i.e., censor “bad” data; This is the same as Model B, except with the tails of the biologically damaging UV radiation distribution removed]
				solar zenith angle [SZA]
D	−0.80	−1.30	biologically damaging UV radiation ~f (O_3_, CLR^^^, SZA)—CLR is a linear function between 40% and 100% clear skies	total column ozone [O_3_]
				CLR [assume a linear function in percent clearness {“classes” of CLR} between 40% and 100% clear skies]
				solar zenith angle [SZA]
E	−0.80	−1.28	biologically damaging UV radiation ~f (O_3_/cos[SZA], cos[SZA])	total column ozone [O_3_]/cos(SZA)
				cos(SZA)
				This is known as Beer–Lambert’s Law

## Abbreviations

CCD: charge-coupled device
CFC: chlorofluorocarbon
CLR: percent clear sky radiation
DOI: US Department of the Interior
DOS: disk operating system
DU: Dobson Unit
DUV: biologically damaging UV radiation
EPA: US Environmental Protection Agency
GMT: Greenwich Mean Time
IAG: Interagency Agreement
LCZA: natural logarithm of the cosine of the solar zenith angle
LDUV: natural logarithm of the biologically damaging UV radiation
LOZ: natural logarithm of the total column ozone
PCLR: fraction of clear sky radiation
NASA: National Aeronautics and Space Administration
NERL: EPA National Exposure Research Laboratory
NIST: US National Institute for Standards and Technology
NOAA: National Oceanographic and Atmospheric Administration
NUVMC: National UV Monitoring Center
NPS: US National Parks Service
O_3_: total column ozone
ORD: EPA Office of Research and Development
RAF: Radiation Amplification Factor
SZA: Solar Zenith Angle
TOMS: NASA Total Ozone Mapping Spectrometer
UGA: University of Georgia at Athens
US: United States
UV: ultraviolet
UVR: ultraviolet radiation

## References

[1] Gerstl SAW, Zardecki A, Wiser HL (1981). Biologically damaging radiation amplified by ozone depletions. Nature.

[2] Smertenko P, Stepanov V, Ol’khovik C, Durzan DJ, Blume Y, Durzan DJ, Smertenko P (2006). New approach to a radiation amplification factor. Cell Biology and Instrumentation: UV Radiation, Nitric Oxide, and Cell Death in Plants.

[3] Parisi AV, Sabburg J, Kimlin MG (2004). Scattered and Filtered Solar UV Measurements.

[4] Madronich S, McKenzie RL, Bjorn LO, Caldwell MM (1998). Changes in biologically active ultraviolet radiation reaching the Earth’s surface. J Photochem Photobiol B.

[5] Pfister G, McKenzie RL, Liley JB, Thomas A, Forgan BW, Long CN (2003). Cloud coverage based on all-sky imaging and its impact on surface solar irradiance. J Appl Meteorol Clim.

[6] Hall ES, Kotowski F (2009). Ground-Based Measurement of Solar Ultraviolet Radiation. McGraw-Hill Yearbook of Science and Technology.

[7] Sabburg J (2001). EPA/UGA, UV/QA Data Correction Project Specification (v2.3).

[8] Weatherhead EC, Frederick JE Report on Geographic and Seasonal Variability of UV Affecting Human and Ecological Health.

[9] Lenoble J (1993). Atmospheric Radiation Transfer.

[10] Warhaft Z (1997). An Introduction to Thermal-Fluid Engineering: The Engine and the Atmosphere.

[11] Frederick JE, Snell HE, Haywood EK (1989). Solar ultraviolet radiation at the earth’s surface. Photochem Photobiol.

[12] Crist KC, Carmichael GR, John K (1994). UV-B exposure and atmospheric ozone: Evaluation of radiative flux to changes in ambient ozone levels. J Hazard Mater.

[13] Sivasakthivel T, Reddy KSK Ozone Layer Depletion and Its Effects: A Review.

[14] Rowland FS (2006). Stratospheric Ozone Depletion. Philos T Roy Soc B.

[15] McCulloch A, Midgley PM, Ashford P (2003). Releases of refrigerant gases (CFC-12, HCFC-22, and HFC-134a) to the atmosphere. Atmos Environ.

[16] United Nations Environment Programme (UNEP) The 1987 Montreal Protocol on Substances that Deplete the Ozone Layer.

[17] US Environmental Protection Agency (US EPA) Clean Air Act Title VI—Stratospheric Ozone Protection.

[18] Brewer MKIV Spectrophotometer Operator’s Manual.

[19] Binyamin J, Davies J, McArthur B (2011). Validation of spectral and broadband UV-B (290–325 nm) irradiance for Canada. Atmos Clim Sci.

[20] Feister U, Cabrol N, Häder D (2015). UV enhancements by scattering of solar radiation from clouds. Atmosphere.

[21] ITACA The Sun as a Source of Energy, Part 4: Irradiation Calculations.

[22] El-Nouby AM (2010). Effect of stratospheric ozone in UVB solar radiation reaching the Earth’s surface at Qena, Egypt. Atmos Pollut Res.

[23] Varotsos C (2005). Airborne measurements of aerosol, ozone, and solar ultraviolet irradiance in the troposphere. J Geophys Res Atmos.

[24] Mckenzie RL, Bjorn LO, Bais A, Ilyas M (2003). Changes in biologically active ultraviolet radiation reaching the Earth’s surface. Photochem Photobiol Sci.

[25] McKenzie RL, Aucamp PJ, Bais AF, Björn LO, Ilyas M (2007). Changes in biologically active ultraviolet radiation reaching the Earth’s surface. Photochem Photobiol Sci.

[26] Ziemke JR, Chandra S, Herman J, Varotsos C (2000). Erythemally weighted UV trends over northern latitudes derived from Nimbus 7 TOMS measurements. J Geophys Res Atmos.

[27] Massen F Computing the Radiation Amplification Factor RAF Using a Sudden Dip in Total Ozone Column Measured at Diekirch, Luxembourg.

